# Skin physiologically based pharmacokinetic modeling: current research progress, software comparison, and future perspectives

**DOI:** 10.1080/10717544.2026.2726638

**Published:** 2026-09-07

**Authors:** Yafen Li, Yuxiao Li, Ya-xin Liu, Han-feng Zhang, Shengnan Zhang, Yun Kuang, Qi Pei, Guoping Yang

**Affiliations:** a Center of Clinical Pharmacology, The Third Xiangya Hospital, Central South University, Changsha, China; b XiangYa School of Pharmaceutical Sciences, Central South University, Changsha, China

**Keywords:** Transdermal and topical delivery systems, physiologically based pharmacokinetic (PBPK), percutaneous absorption, quantitative structure-activity relationship (QSAR) models, digital twin

## Abstract

Transdermal and topical delivery systems (TDS) have emerged as the third-largest route of administration due to their ability to bypass first-pass metabolism and maintain stable plasma concentrations. Skin physiologically based pharmacokinetic (PBPK) models mechanistically characterize the percutaneous absorption process, predict local and systemic exposure, and enhance research and development efficiency through virtual clinical trials. This paper first elaborates on the theoretical foundation of skin PBPK modeling based on Fick's second law of diffusion and compares the model assumptions and application scenarios of four well-established and validated modeling platforms: Simcyp, GastroPlus, Skin-CAD, and PK-Sim. The findings indicate that Simcyp provides a refined depiction of skin physiological structure and is widely applied in clinical evaluation and special population prediction. GastroPlus focuses more on the mechanistic description of TDS formulations. Skin-CAD, as a specialized software, is extensively utilized for *in vitro–in vivo* extrapolation (IVIVE) predictions and formulation optimization. PK-Sim, as a freely accessible open-source software, has been used to investigate the physiological ontogeny of skin in pediatric populations. However, existing studies share limitations such as highly inconsistent model structures with varying complexity, challenges in acquiring accurate parameters, and a scarcity of physiological information for special populations. Future research should prioritize optimizing parameter acquisition methods and broadening physiological databases for pediatric, geriatric, and skin-lesion populations. Additionally, integrating non-invasive imaging technologies to construct high-precision ‘digital twin’ skin models represents a critical future direction. These advancements will drive the transition of PBPK models from descriptive tools to supportive instruments for individualized dosing and regulatory decision-making.

## 1. Introduction

**Figure 1.**
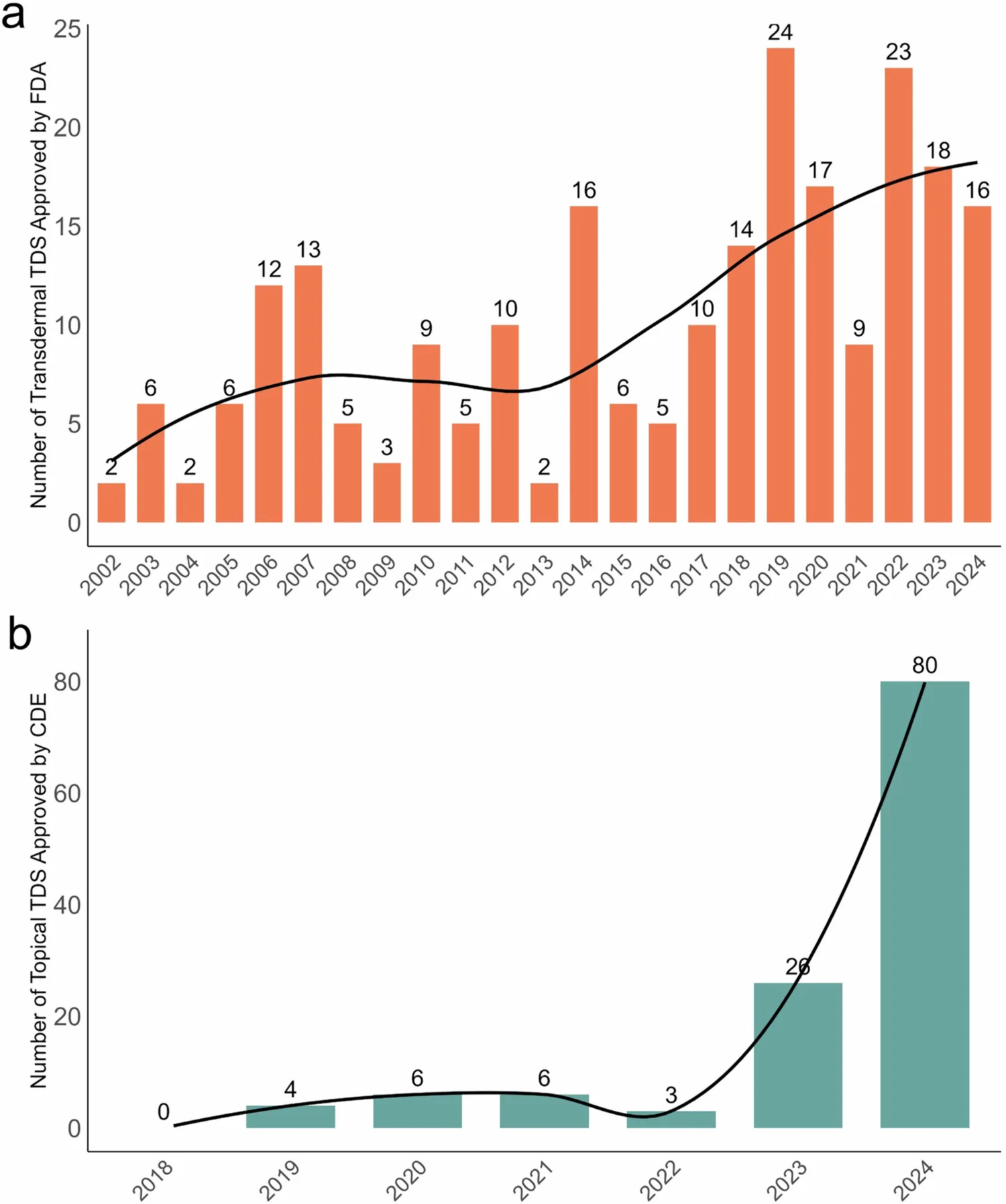
Transdermal TDS products approved by the FDA (a) and topical TDS products approved by the CDE (b). (Data are sourced from the FDA Orange Book (U.S. Food and Drug Administration 2025) and CDE registration data (NMPA 2025)).

Transdermal and topical delivery systems (TDS) have emerged as the third-largest drug delivery category following oral and injectable administration. This prominence is attributed to their ability to bypass first-pass metabolism, maintain stable plasma concentrations through controlled drug release, and enhance patient compliance (Prausnitz and Langer 2008; Ramadon et al. 2022). Based on their therapeutic objectives, the TDS are categorized into transdermal delivery systems (for systemic effects) and topical delivery systems (for local effects). In recent years, the market prospects for TDS have shown a continuous and promising upward trend. This is reflected in the steady increase in the number of transdermal products approved by the U.S. Food and Drug Administration (FDA) (U.S. Food and Drug Administration 2025) and topical products approved by the Center for Drug Evaluation (CDE) in China (NMPA 2025) (Figure 1). As of October 2025, the U.S. FDA had approved 22 drugs for transdermal delivery covering 16 therapeutic indications. These approved products are primarily categorized within the fields of analgesia and neuropsychiatry (Table 1).

**Table 1.** Systemically transdermal TDS drugs approved by the U.S. FDA.

Drug	Formulation	Indication	Company	Approval year
Scopolamine	Patch	Motion sickness	Baxter Healthcare Corp.	1982
Clonidine	Patch	Hypertension	Lavipharm SA	1984
Nitroglycerin	Ointment	Angina pectoris	Fougera Pharmaceuticals	1988
Estradiol	Extended-release film	Menopausal symptoms	Bayer	1994
Nicotine	Extended-release film	Smoking cessation	Sanofi	1996
Estradiol/Norethindrone acetate	Extended-release film	Menopausal symptoms	Noven	1998
Testosterone	Gel	Testosterone deficiency	Besins Healthcare	2000
Estradiol/Levonorgestrel	Extended-release film	Menopausal symptoms	Bayer	2003
Oxybutynin	Extended-release film	Overactive bladder	Allergan	2003
Fentanyl	Extended-release film	Chronic pain	Mylan Technologies	2005
Methylphenidate	Extended-release film	ADHD	Noven	2006
Selegiline	Extended-release film	Depression	Somerset Pharmaceuticals	2006
Lidocaine hydrochloride	Patch	Post-herpetic neuralgia	Powder Pharmaceuticals	2007
Rivastigmine	Extended-release film	Mild-to-moderate Alzheimer's disease	Sandoz	2007
Rotigotine	Extended-release film	Parkinson's disease	Union Chimique Belge	2007
Granisetron	Extended-release film	Chemotherapy-induced nausea and vomiting	Cumberland Pharmaceutical	2008
Buprenorphine	Extended-release film	Severe and persistent pain	Purdue Pharma	2010
Ethinyl estradiol/Etonogestrel	Extended-release film	Contraception	Mylan Technologies	2014
Asenapine	Patch	Schizophrenia	Hisamitsu Pharmaceutical	2019
Ethinyl estradiol/Levonorgestrel	Patch	Contraception	Agile Therapeutics	2020
Dexmethylphenidate	Patch	ADHD	Noven	2022
Donepezil hydrochloride	Patch	Alzheimer's disease	Corium LLC	2022

Note: Data are sourced from the FDA Orange Book (FDA, 2025). Only the first approved product for each active ingredient is presented. ADHD, attention deficit hyperactivity disorder.

Despite their advantages, the drug development process for TDS is often hindered by the inherent complexity of the formulations (Leveque et al. 1980) and the intricate factors influencing skin absorption (Dąbrowska et al. 2018). Notably, for topical delivery systems, the development process is further complicated by the significant challenges associated with sampling local pharmacokinetics (PK) and the low sensitivity of clinical efficacy evaluations. In this context, skin physiologically-based pharmacokinetic (PBPK) models, which integrate physiological parameters and drug properties, provide a valuable solution. Skin PBPK models utilize mathematical equations to mechanistically characterize the release of drugs from TDS formulations, their percutaneous absorption, and their subsequent distribution and elimination within the systemic circulation, while incorporating key physiological factors that influence PK (Grimstein et al. 2019; Zhao et al. 2019). By employing skin PBPK models, researchers can effectively simulate the dynamic PK profiles of both local and systemic exposure following TDS administration to assess whether the target exposure is achieved. Ultimately, this approach serves to accelerate new drug development and facilitate the consistency evaluation of generic drugs.

Furthermore, the U.S. FDA has published regulatory considerations for the use of PBPK models in supporting bioequivalence (BE) studies for TDS products. The agency has also provided funding to advance the development of skin PBPK tools and advocated for the use of virtual clinical studies to accelerate the submission and approval of generic drugs (Tsakalozou et al. 2022). A significant regulatory milestone was achieved in 2020, when Tsakalozou et al. utilized the Multi-Phase Multi-Layer Mechanistic Dermal Absorption (MPML MechDermA) model to support the successful reduction of clinical BE study requirements for a generic diclofenac sodium gel (Tsakalozou et al. 2021). Currently, the predominant PBPK models in this field include the MPML MechDermA model within Simcyp (Patel et al. 2022), the Transdermal Compartmental Absorption & Transit (TCAT) model in GastroPlus (van Osdol et al. 2024), the specialized Skin-CAD software developed by Biocom Systems (Nakamura et al. 2012), and the Skin Permeation Model (SPM) integrated into PK-Sim/MoBi (Hamadeh and Sevestre 2013).

Nevertheless, most existing skin PBPK models are commercial, non-open-source platforms, each employing distinct construction methodologies. Given this current landscape, the present study provides a comprehensive review of the percutaneous absorption process and its influencing factors, followed by a comparative analysis of the modeling theories and research cases associated with various skin PBPK platforms. This work aims to provide readers with a thorough understanding of the development logic and underlying assumptions of skin PBPK models, while exploring potential optimization strategies and future research directions to further facilitate the development and application of skin PBPK.

## 2. Skin physiological structure and the percutaneous absorption process

**Figure 2.**
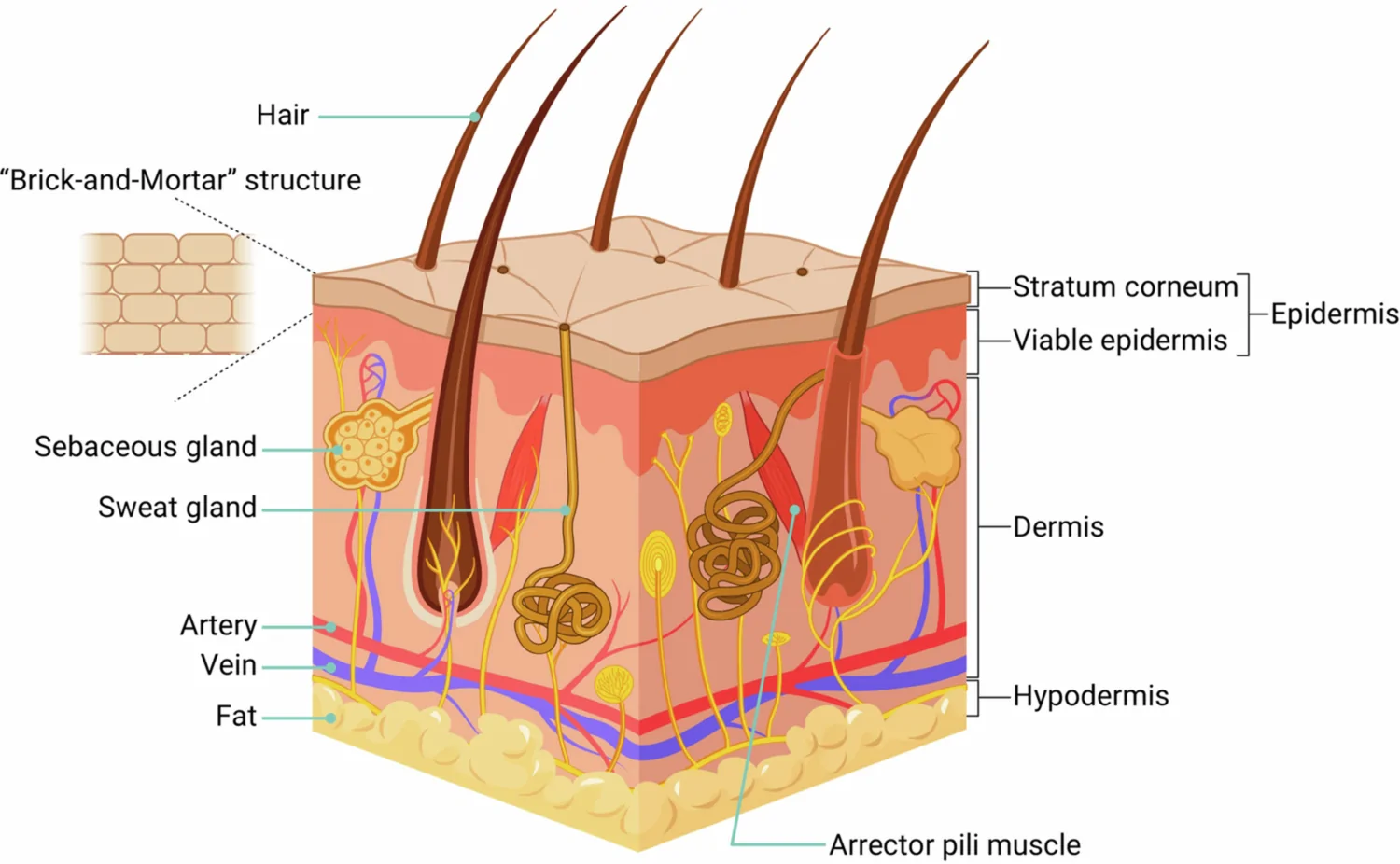
Schematic representation of skin physiological structure.

As shown in Figure 2, the skin is composed of the epidermis, dermis, hypodermis, and appendages, such as sebaceous or sweat glands, that penetrate the epidermis and dermis. The epidermis is further divided into the non-viable stratum corneum (SC) and the viable epidermis (VE). Located at the outermost layer of the skin, the SC consists of corneocytes embedded within a densely packed extracellular lipid matrix. This configuration of SC is frequently described using the ‘brick-and-mortar’ structural analogy and serves as the primary barrier to drug permeation and absorption (Anissimov et al. 2013).

**Figure 3.**
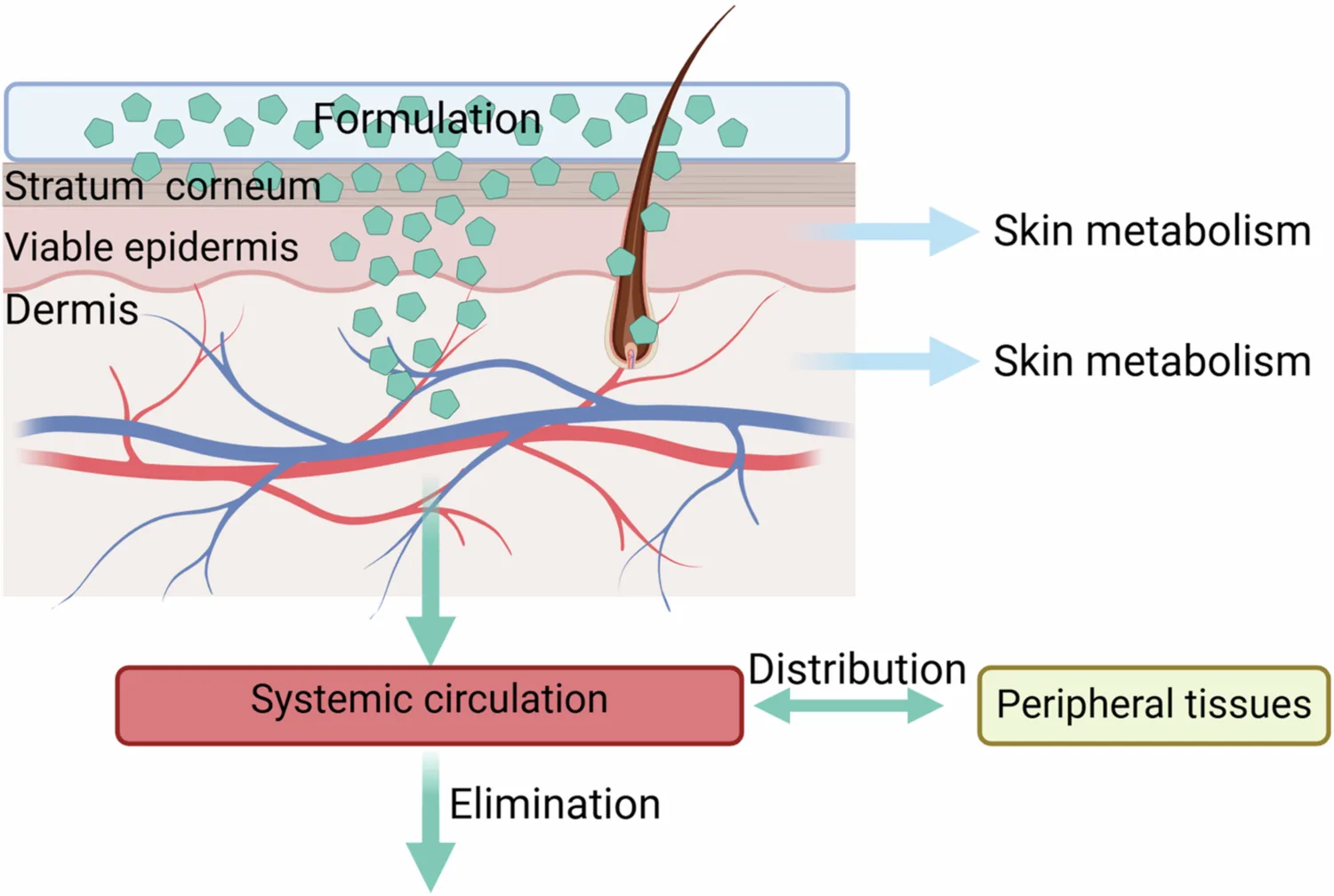
Schematic diagram of the percutaneous drug absorption process.

As illustrated in Figure 3, the absorption process of a drug following TDS administration involves several sequential steps (Jepps et al. 2013). Initially, the drug is released from the formulation and dissolves onto the skin surface, followed by partitioning into the SC. Subsequently, the drug undergoes diffusion through the SC and partitioning into the VE, followed by diffusion through the VE and partitioning into the dermis. Finally, the drug is taken up by the capillary network in the dermis and enters the systemic circulation. Drugs situated near appendages can also be directly absorbed into the dermis and subsequently into the bloodstream. Furthermore, some drugs may experience keratin binding within the SC or metabolic clearance within the VE and dermis (Dancik et al. 2008).

**Figure 4.**
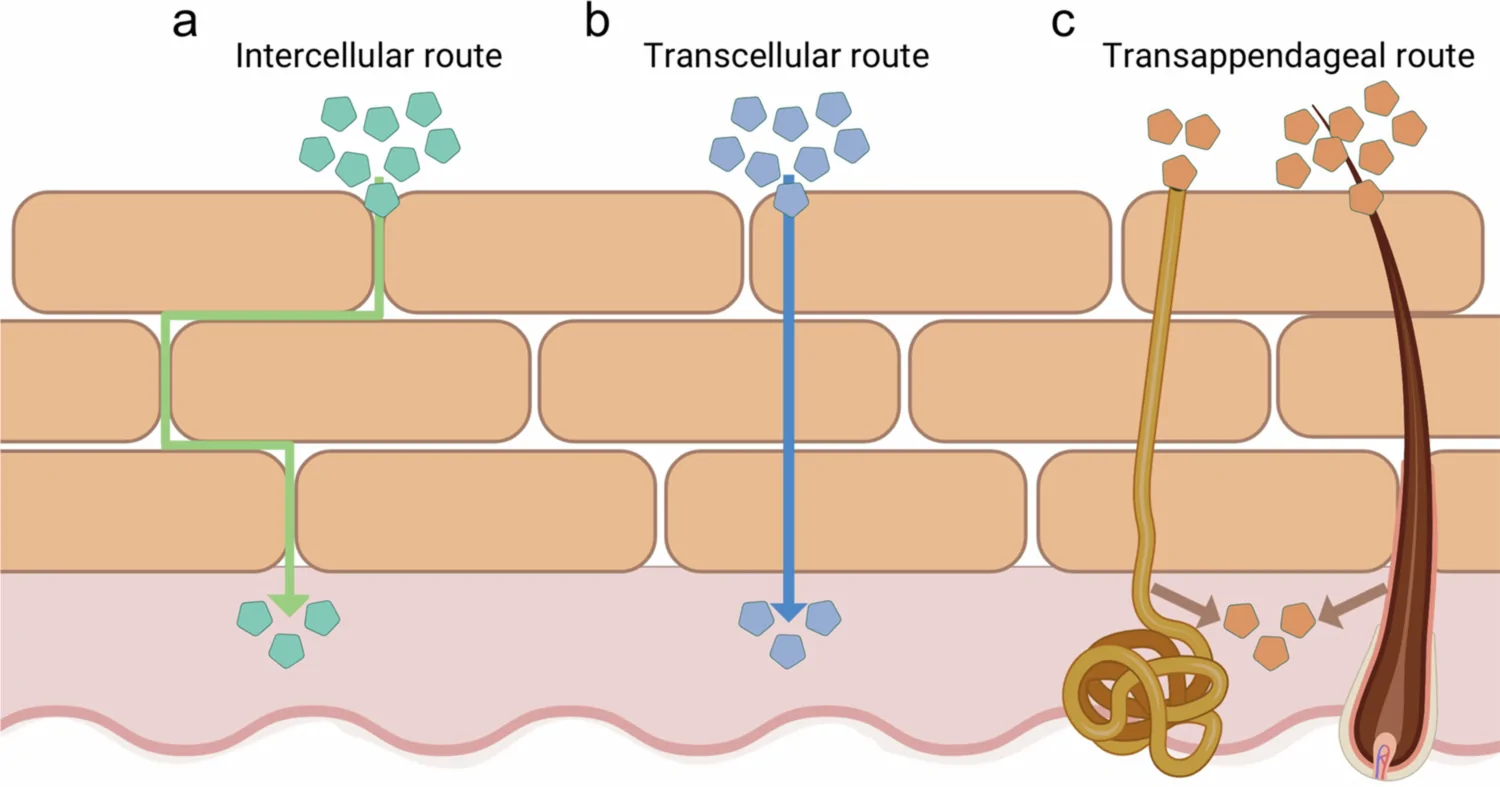
Three pathways of drug penetration through the SC barrier. Pathway (a) represents the intercellular route, (b) the transcellular route, and (c) the transappendageal route.

There are three primary pathways for a drug to penetrate the SC, which acts as the rate-limiting barrier to skin absorption: the intercellular route, the transcellular route, and the transappendageal route (Figure 4) (Desai et al. 2010; Dąbrowska et al. 2018). Hydrophobic drugs primarily utilize the intercellular route by permeating through the lipid matrix between corneocytes, whereas hydrophilic drugs predominantly enter the skin via the transcellular route. The transappendageal route provides a rapid and direct channel through the SC, serving as an effective pathway for ionic compounds and drugs with larger molecular weights. However, because sebaceous and sweat glands occupy only a minimal fraction of the skin surface area, approximately 0.1%, the total amount of drug accessible to this pathway is significantly limited (Dancik et al. 2008).

## 3. Factors influencing percutaneous drug absorption

**Figure 5.**
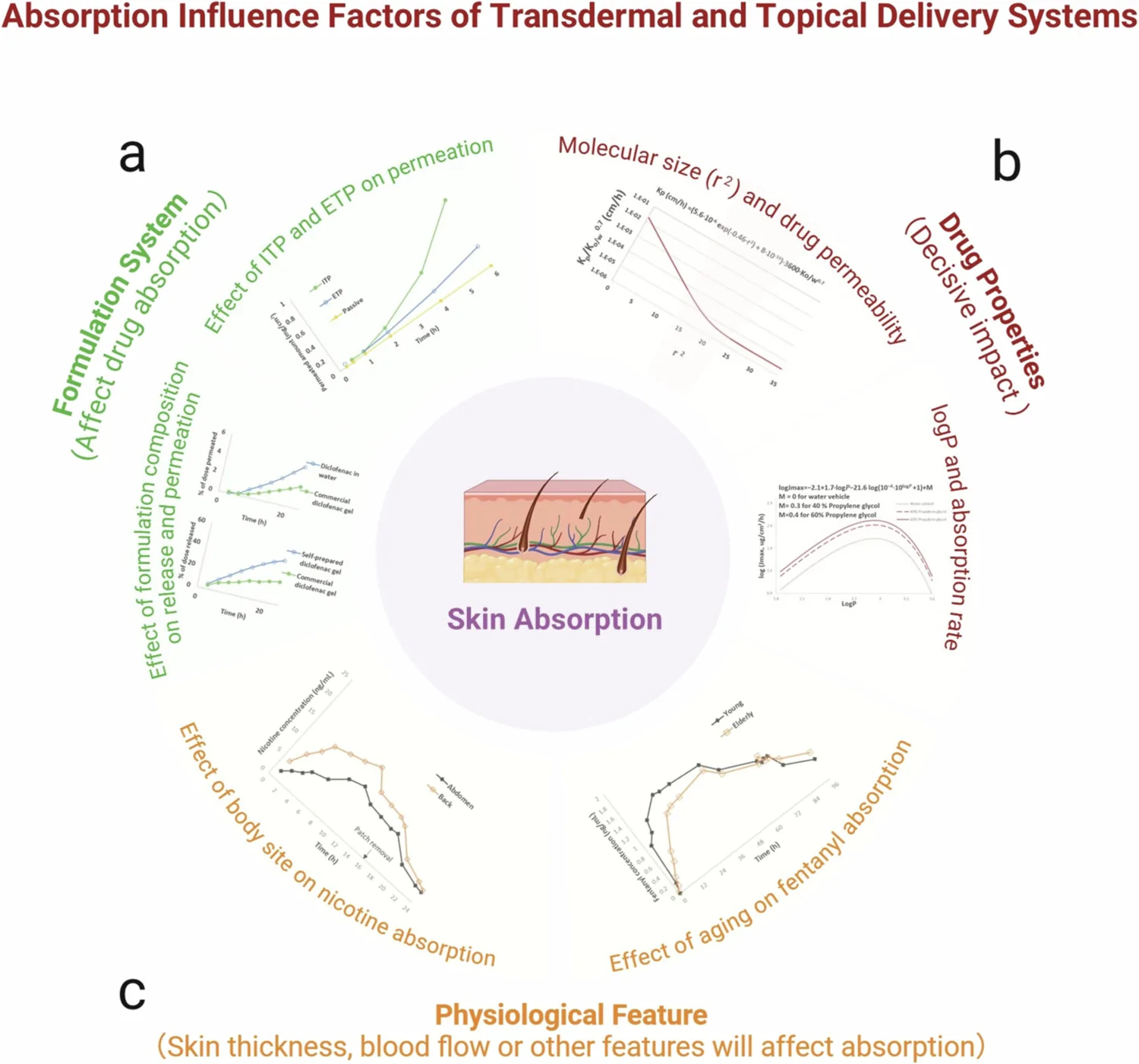
Factors influencing percutaneous drug absorption. (a) Formulation factors: Impact of formulation design on the release and permeation of diclofenac, adapted from (Russo et al. 2020); enhancement of tacrine permeation by iontophoresis (ITP) or electroporation (ETP), adapted from (Hirsch et al. 2004). (b) Drug factors: Molecular size affecting percutaneous permeability, adapted from (Mitragotri 2003); the effect of logP on the percutaneous absorption rate, adapted from (Zhang et al. 2011). (c) Physiological factors: Influence of anatomical sites on nicotine absorption, adapted from (Dancik et al. 2008); effect of age on fentanyl absorption, adapted from (Thompson et al. 1998).

As illustrated in Figure 5, the factors influencing the percutaneous absorption of TDS primarily encompass the physicochemical properties of the drug, the formulation system, and physiological factors. Physicochemical properties, such as molecular weight (MW) and the logarithm of octanol-water partition coefficient (K_o/w_) (LogP), significantly impact the partitioning and diffusion of a drug within the skin, thereby determining the rate and extent of percutaneous absorption (Potts and Guy 1992; Wang et al. 2007; Zhang et al. 2011). Regarding the formulation, various dosage forms including patches, gels, and emulsions modulate the drug release profile. Furthermore, the addition of chemical penetration enhancers to a formulation can increase the thermodynamic activity of the drug and reduce skin resistance, while advanced delivery techniques such as microneedles promote permeation by physically disrupting the structure of the SC, and iontophoresis actively drives drug transport across the skin via electromigration and electroosmosis (Aboofazeli et al. 2002; Hirsch et al. 2004; Williams and Barry 2004; Russo et al. 2020; Kushwaha and Palei 2024). In terms of physiological factors, such as skin thickness, hydration level, cutaneous blood flow, and hair follicle density are key factors of drug absorption (Dąbrowska et al. 2018). These physiological features can be altered by anatomical site, age, and pathological skin conditions, such as dermatitis or eczema, thereby influencing the PK following TDS administration (Leveque et al. 1980; Ya-Xian et al. 1999; Sandby-Møller et al. 2003; Halling-Overgaard et al. 2017). Additionally, factors such as sex, smoking habits, body weight, and ethnicity are characterized by distinct skin physiological features, but their specific effects on percutaneous absorption have yet to be effectively validated through clinical PK studies (Dąbrowska et al. 2018).

## 4. Theoretical foundations of skin PBPK model

### 4.1. Fick's first law characterizing steady-state permeation

The steady-state permeation of a drug through the skin can be described by Fick's first law (Equation (1)), which states that the steady-state flux ( $J_{\mathrm{ss}}$ ) per unit area (A) is defined by the concentration gradient ( $\Delta C=C_{0}-C_{i}$ ) across the upper and lower boundaries of the skin, the diffusion coefficient (D), and the skin thickness (h) (Anissimov et al. 2013; Tsakovska 2017).

(1) $$J_{\mathrm{ss}}=\frac{Q}{A\;∙\;(t-t_{\mathrm{lag}})}=\frac{D\;∙\;\Delta C}{h}$$

In Equation (1), Q represents the cumulative amount of drug permeated at time t, and $t_{\mathrm{lag}}$ is the lag time, defined as the intercept on the time axis when the linear portion of the permeation curve with slope $J_{\mathrm{ss}}$ is extrapolated.

Once the drug enters the dermis, it is rapidly cleared by cutaneous capillaries, maintaining a near-zero concentration at the lower boundary of the skin (vascular side) compared to the upper boundary (dosing side), which is regarded as perfect sink conditions. To simulate the *in vivo* percutaneous absorption process, *in vitro* skin permeation tests (IVPT) generally utilize a receptor medium where the drug concentration is maintained well below its saturation solubility. Under these sink conditions, the concentration difference ΔC approximately equals the concentration at the upper boundary of the stratum corneum ( $C_{0}$ ). This boundary concentration $C_{0}$ is related to the drug concentration in the vehicle ( $C_{v}$ ) through the partition coefficient K, expressed as $C_{0}=K\;∙\;C_{v}$ . Consequently, the calculation of $J_{\mathrm{ss}}$ in Equation (1) can be transformed into Equation (2). By integrating the skin thickness h with the parameters D and K, where both are determined by drug properties, the permeability coefficient ( $k_{p}$ ) can be defined as $k_{p}=D\;∙\;K/h$ , allowing for the further simplification shown in Equation (3) (Hansen et al. 2012; Jepps et al. 2013; Tsakovska 2017).

(2) $$J_{\mathrm{ss}}=\frac{D\;∙\;K{\;∙\;c}_{v}}{h}$$

(3) $$J_{\mathrm{ss}}=k_{p}\;∙\;c_{v}$$

When an infinite dose is applied, the drug concentration within the skin reaches its maximum solubility ( $S_{0}$ ), resulting in the maximum flux ( $J_{\max}$ ), which is calculated using Equation (4).

(4) $$J_{\max}=\frac{D\;∙\;S_{0}}{h}$$

Based on $k_{p}$ , the maximum solubility in the vehicle ( $S_{v}$ ), and the partition coefficient $K_{\mathrm{sc}:v}$ , $J_{\max}$ can be determined by Equation (5).

(5) $$J_{\max}=\frac{D\;∙\;K\;∙\;S_{v}}{h}=k_{p}\;∙\;S_{v}$$

Utilizing IVPT experimental results, numerous researchers have developed various quantitative structure-permeability relationship (QSPR) models to predict $k_{p}$ , $J_{\mathrm{ss}}$ , or $J_{\max}$ based on drug physicochemical properties. These QSPR models characterize the rate and extent of percutaneous absorption. Equation (6) illustrates the QSPR model reported by Potts and Guy, which predicts the permeability coefficient $k_{p}$ using the MW and K_o/w_ (Potts and Guy 1992).

(6) $$\log\;k_{p}(\mathrm{cm}/s)=0.71\;∙\;\log\;K_{o/w}-0.0061\;∙\;\mathrm{MW}-6.3$$

### 4.2. Fick's second law characterizing the spatiotemporal dynamic permeation

Fick's first law and QSPR models rely on the assumptions of sink conditions and steady-state kinetics. However, the percutaneous absorption process in the human body does not consistently adhere to the assumption of perfect sink conditions. Furthermore, the drug concentration in the delivery vehicle changes as the drug is absorbed, making it impossible for these steady-state models to characterize the dynamic changes in drug concentration across both time and space within the skin. In contrast, Fick's second law (Equation 7) can dynamically characterize the diffusion processes of drugs within each individual skin layer at both temporal and spatial levels, and is thus widely utilized in the construction of skin PBPK models (Gu et al. 2020). Fick's second law assumes that drug transport through the skin occurs via passive diffusion, with the rate determined by the diffusion coefficient (D) and the driving force of the drug concentration gradient. Because different skin layers exert distinct resistance to drug transport, each layer is assigned a specific value of D. In addition, to characterize interfacial partitioning, it is necessary to define the boundary conditions between the formulation vehicle and SC, as well as between adjacent skin layers (e.g. the SC and the VE), which must comply with the partition law (Equation 8) and the law of conservation of mass (Equation 9).

(7) $$\frac{\partial C(x,t)}{\partial t}=D\frac{\partial^{2}C(x,t)}{\partial x^{2}}$$

(8) $$\frac{C_{1}}{C_{2}}=K_{1:2}$$

(9) $$D_{1}\frac{\partial C_{1}(h,t)}{\partial x}=D_{2}\frac{\partial C_{2}(h,t)}{\partial x},x=h$$

where $C_{1}$ and $C_{2}$ represent the drug concentrations on either side of the adjacent boundary. $K_{1:2}$ denotes the partition coefficient between adjacent skin layers or between the formulation and the outermost skin. $D_{1}$ and $D_{2}$ represent the diffusion coefficients of the two adjacent skin layers, or of the formulation and its adjacent skin layer, respectively.

### 4.3. Development of skin PBPK model

#### 4.3.1. Structural hypotheses of skin PBPK model

PBPK models typically employ ordinary differential equations (ODEs), based on the well-stirred model assumption (Equation (10)), to describe mass exchange and dynamic changes in drug concentration between the systemic circulation and various organs (Dong and Park 2018). In contrast, the percutaneous absorption process characterized by Fick's second law is defined by partial differential equations (PDEs). To effectively link the skin absorption module with the systemic circulation module within a PBPK framework, the PDEs of Fick's second law must be discretized into a series of ODEs.

**Figure 6.**
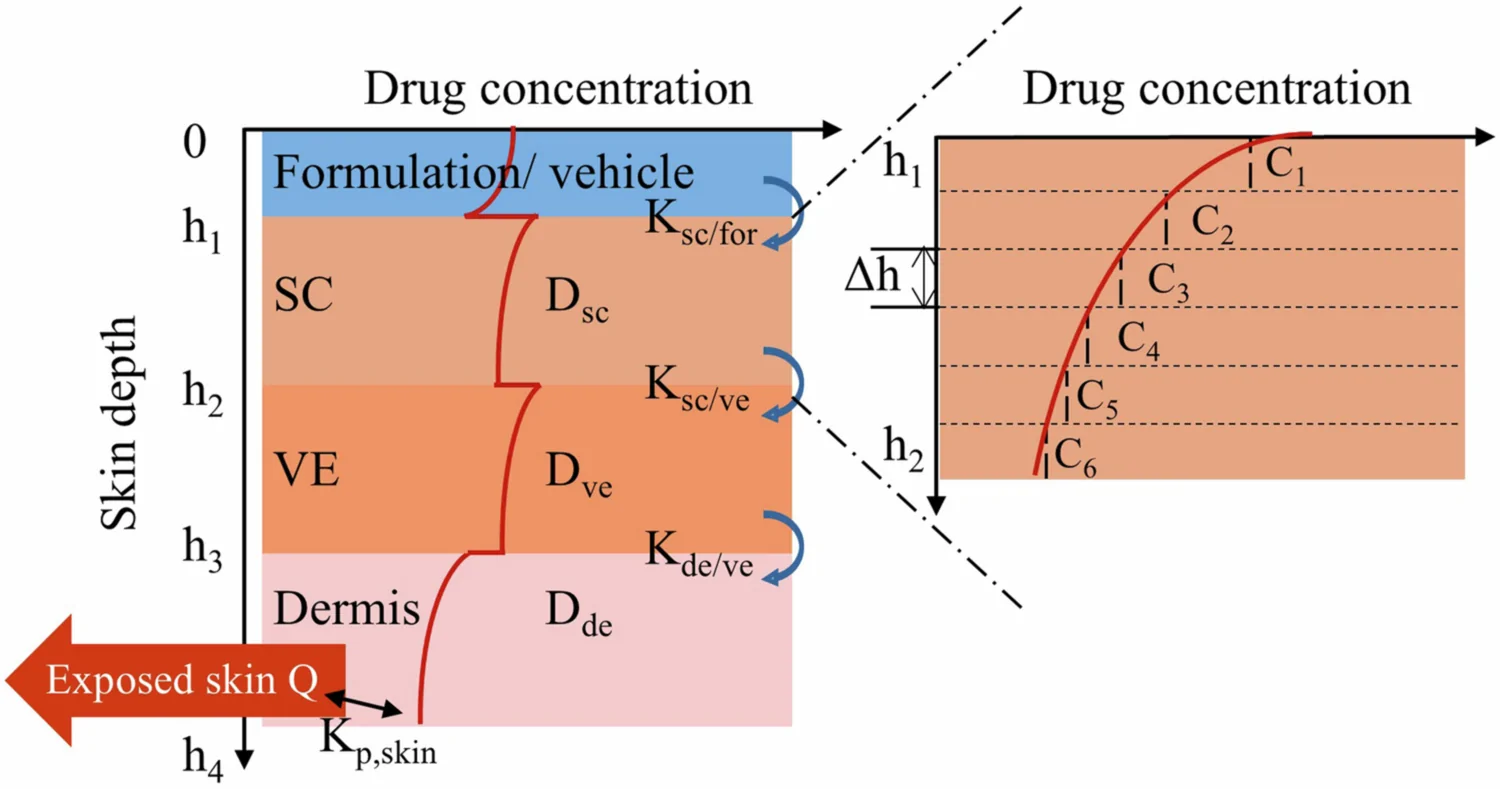
Schematic of the discretization of skin structure using the finite difference method. The diagram illustrates the SC layer discretized into six compartments as an example, assuming each compartment is well-mixed with a uniform concentration distribution represented by C_1_ to C_6_. K_sc/for_, K_sc/ve_, and K_de/ve_ represent the partition coefficients for SC/formulation (or vehicle), SC/viable epidermis, and dermis/viable epidermis, respectively. D_sc_, D_ve_, and D_de_ denote the diffusion coefficients within the SC, viable epidermis, and dermis, respectively.

The finite difference method (FDM) discretizes the SC, VE, dermis, and subcutaneous layers into multiple sub-layers. This method assumes that each discretized sub-compartment is well-stirred and maintains a uniform (or highly similar) concentration, which is a common approach for obtaining approximate solutions to PDEs (Hu et al. 2023). While increasing the number of discretized layers improves computational accuracy, it also significantly raises the computational load of the model. The structural diagram of the skin discretization process is shown in Figure 6. Specifically, as shown in Equation (11), the change in drug amount per unit time within the *i*-th skin compartment is defined by the influx rate from the preceding layer and the efflux rate from the current layer. Furthermore, the discontinuous concentration changes between adjacent skin are defined by the partition law using the partition coefficient $K_{i:i-1}$ or $K_{i+1:i}$ . Finally, Equation (12) defines the process by which the drug is absorbed from the dermis into the systemic circulation through mass exchange. The blood flow to the exposed skin ( $Q_{\mathrm{skin},\exp}$ ) is determined by the ratio of the application area (A) to the total body surface area (BSA), multiplied by the total skin blood flow ( $Q_{\mathrm{skin},\mathrm{total}}$ ) (Equation (13)).

(10) $$\frac{dC_{T}}{\mathrm{dt}}=\frac{Q_{T}}{V_{T}}\;\cdot \;(C_{a}-\frac{C_{T}}{K_{p}})$$

(11) $$\frac{dM_{i}}{\mathrm{dt}}=\left(\frac{D_{i}\;\cdot \;A}{H_{i}}\left(\left(\frac{K_{i:i-1}\;\cdot \;M_{i-1}}{V_{i-1}}\right)-\left(\frac{M_{i}}{V_{i}}\right)\right)\right)-\left(\frac{D_{i+1}\;\cdot \;A}{H_{i+1}}\left(\left(\frac{K_{i+1:i}\;\cdot \;M_{i}}{V_{i}}\right)-\left(\frac{M_{i+1}}{V_{i+1}}\right)\right)\right)$$

(12) $$\frac{dM_{i}}{\mathrm{dt}}=\left(\frac{D_{i}\;\cdot \;A}{H_{i}}\left(\left(\frac{K_{i:i-1}\;\cdot \;M_{i-1}}{V_{i-1}}\right)-\left(\frac{M_{i}}{V_{i}}\right)\right)\right)+Q_{\mathrm{skin},\exp}\cdot \left(C_{a}-\frac{M_{i}}{K_{p,\mathrm{skin}}\;\cdot \;V_{i}}\right)$$

(13) $$Q_{\mathrm{skin},\exp}=\frac{Q_{\mathrm{skin},\mathrm{total}}\;\cdot \;A}{\mathrm{BSA}}$$

where $C_{T}$ represents the tissue drug concentration. $Q_{T}$ denotes the tissue blood flow. $V_{T}$ is the tissue volume. $C_{a}$ is the arterial blood concentration. $K_{p}$ is the tissue-to-venous blood partition coefficient. $D_{i}$ and $D_{i+1}$ represent the diffusion coefficients of the i-th and the subsequent discretized skin compartments, respectively. $H_{i}$ and $H_{i+1}$ represent the thickness of the i-th and the subsequent compartments. $\frac{M_{i}}{V_{i}}$ , $\frac{M_{i-1}}{V_{i-1}}$ , and $\frac{M_{i+1}}{V_{i+1}}$ represent the drug concentrations in the i-th, preceding, and subsequent compartments, respectively. $K_{p,\mathrm{skin}}$ represents the skin-to-venous blood partition coefficient.

#### 4.3.2. Mathematical hypotheses of TDS formulation

**Figure 7.**
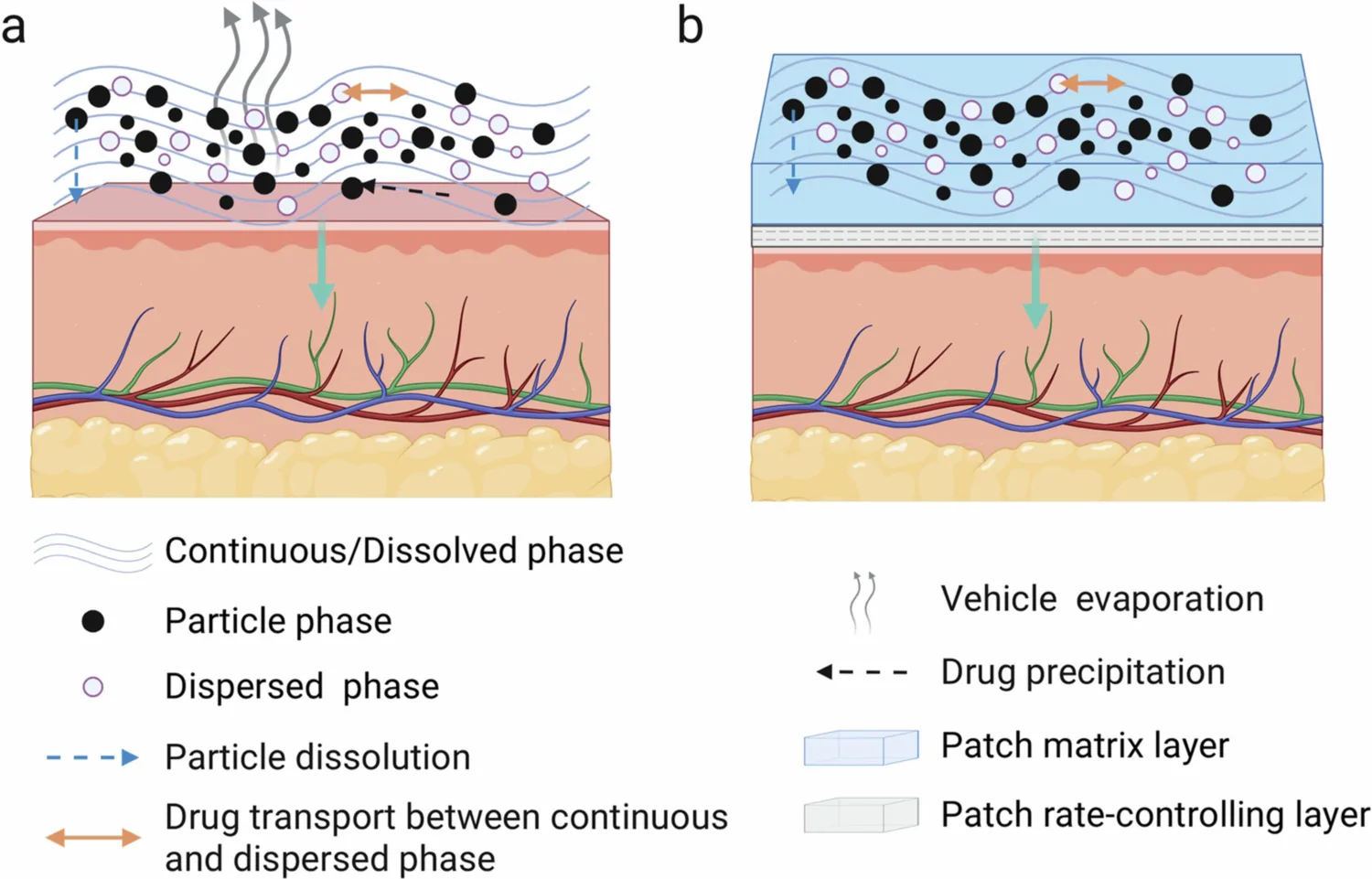
Schematic representation of structural hypotheses for formulation systems. (a) liquid/semi-solid formulation, (b) patch formulation system.

As illustrated in Figure 7, current research typically defines TDS formulations as multiphase systems comprising continuous/dissolved, dispersed, and solid particle phases(Paul 2010; Siepmann and Peppas 2011; Patel et al. 2022). Based on formulation properties, skin PBPK models adopt the assumption of single-phase, biphasic, or triphasic configurations to mathematically characterize the processes of drug dissolution, multiphase transition, and release (Wang and Flanagan 1999; Mori and Tojo 2006; Hamadeh and Sevestre 2013; Patel et al. 2022; van Osdol et al. 2024). Single-phase systems represent homogeneous, fully dissolved solutions or gels, involve only drug release from the vehicle to the skin. Biphasic systems include suspensions or ointments (continuous and solid phases) and emulsions (dispersed and continuous phases). The former involves particle dissolution before release, while the latter focuses on the partitioning between the two phases and the subsequent release of the drug. Triphasic systems, such as creams containing solid particles, are the most complex because they involve simultaneous partitioning, dissolution, and release mechanisms. The assumption of multiphase systems is also applicable to the drug matrix layer of patches, although they are predominantly characterized as homogeneous single-phase systems. Furthermore, for patches incorporating a rate-controlling layer and/or an adhesive layer, one to two additional formulation compartments may be integrated based on the specific product design (Figure 7b). These additional layers mechanistically describe drug partitioning and diffusion from the matrix into the rate-controlling and adhesive layers, followed by subsequent release into the SC, processes that remain governed by Fick's second law of diffusion and the partition law (Mori and Tojo 2006; Han et al. 2022).

Furthermore, some TDS products contain significant quantities of evaporating solvents, such as water and ethanol. The evaporation of these solvents increases drug concentration, which alters the thermodynamic activity and driving force of the formulation (Grice et al. 2010; Gajjar et al. 2013). If the concentration exceeds saturation, the drug may precipitate, thereby changing the rate and extent of drug absorption (Figure 7a). Evaporation rates can be determined experimentally through evaporation-time curves (Ramezanli et al. 2021) or predicted using established mathematical models (Gajjar et al. 2013). Unlike liquid or semi-solid formulations, patches feature a backing layer that prevents solvent loss; consequently, solvent evaporation and subsequent drug precipitation generally do not need to be considered during model construction (Figure 7b). Additionally, a first-order sedimentation model is commonly used to describe the drug precipitation process (Pathak et al. 2017).

#### 4.3.3. Sources of key parameters for skin PBPK modeling

As indicated by Equation (11), the critical parameters for the percutaneous absorption module are the diffusion coefficients (D) within various skin layers including the SC, VE, and dermis, along with the partition coefficients (K) between these layers. For a specific drug, conducting IVPT study using excised human skin is regarded as the ‘gold standard’ for obtaining these parameters, which can be calculated or fitted based on Fick's second law of diffusion (Equations 14–15) (Tojo et al. 1987; Hansen et al. 2012; Jepps et al. 2013; Han et al. 2022). However, human skin is expensive and scarce, and most drugs lack reported IVPT data. Compounding these challenges, determining parameters D and K from IVPT experiments is difficult because t_lag_ measurements are imprecise (Hoang 1992).

(14) $$D=\frac{h^{2}}{6\;\cdot \;t_{\mathrm{lag}}}$$

(15) $$K=\frac{J_{\mathrm{ss}}*h}{D*C_{v}}$$

To improve efficiency, quantitative structure-activity relationship (QSAR) models are frequently employed to predict these parameters based on physicochemical properties, such as molecular size and $K_{o/w}$ (Mitragotri 2003; Wang et al. 2007). This study summarizes currently available QSAR models and their respective development methodologies in Table 2. As demonstrated in Table 2, the source data utilized for QSAR model development are frequently restricted, specifically characterized by a narrow range of physicochemical properties or limited sample sizes. For instance, Wang et al. developed a QSAR model (Equation 28) to predict the SC lipid diffusion coefficient (D_sc,lip_) based on lateral diffusion experiments conducted with six drugs in extracted SC lipid (Wang et al. 2007). Furthermore, model development data may originate from inconsistent experimental methodologies and variations in skin species or preparation procedures, such as the use of full-thickness skin, isolated layers, or synthetic membranes. Additionally, it is noteworthy that certain QSAR models are derived entirely from theoretical deductions rather than experimental measurements, such as the model represented by Equation 24.

**Table 2.** Summary of QSAR models for predicting key parameters in skin PBPK modeling.

Parameters	Definition	QSAR equation	Sample size (*N*)	Log $K_{o/w}$ range	Data source and experimental methods	Data type	Species	Source
K_sc/w_	SC/water partition coefficients	$K_{\mathrm{sc}/w}={\left(K_{o/w}\right)}^{0.74}$ (Eq.16)	93	−2.25–5.49	This equation was established by isolating the $K_{o/w}$ -dependent terms from the QSPR model for predicting $K_{p}$ ( ${\log\;K}_{p}\left(\mathrm{cm}/h\right)=0.74∙\;\log\;K_{o/w}-0.006∙\mathrm{MW}-2.8$ , Eq.17) reported by Potts and Guy 1992 (Potts and Guy 1992). The original dataset used for the development of the $K_{p}$ QSAR model was derived from the IVPT dataset compiled by Flynn (1990).	Experimental and derived	Human	(Cleek and Bunge 1993)
		$\log\;K_{\mathrm{sc}/w}={-0.006+0.57∙\;\log\;K_{o/w}}^{\;}$ (Eq.18)	34	−0.26–4.0	Determined from experimental $K_{\mathrm{sc}/w}$ values obtained via SC partition equilibrium assays across three independent studies: Scheuplein 1967, Scheuplein et al. 1969, and Roberts et al. 1977.	Experimental	Human	(Cleek and Bunge 1993)
		$\log\;K_{\mathrm{sc}/w}={-0.024+0.59\;∙\;\log\;K_{o/w}}^{\;}$ (Eq.19)	45	−0.77–5.49	Determined from experimental $K_{\mathrm{sc}/w}$ values obtained via SC partition equilibrium assays across four independent studies: Scheuplein and Blank 1971, Lien and Tong 1973, Anderson et al. 1976, and Roberts 1976.	Experimental	Human	(Roberts et al. 1996)
Ksc,lip/w	SC lipid/water partition coefficients	$K_{\mathrm{sc},\mathrm{lip}/w}={\left(K_{o/w}\right)}^{0.76}$ (Eq.20)	12	1.08–5.49	Derived from two distinct datasets reported by Anderson et al. 1988 and Johnson 1995. 1. Anderson et al. 1988 dataset: $K_{\mathrm{sc},\mathrm{lip}/w}$ was determined via partition equilibrium assays using either extracted pure SC lipids or calculated by subtraction between intact SC and delipidized SC. 2. Johnson 1995 dataset: $K_{\mathrm{sc},\mathrm{lip}/w}$ was determined via partition equilibrium assays using either extracted pure SC lipids or intact SC.	Experimental	Human	(Johnson et al. 1997)
		$K_{\mathrm{sc},\mathrm{lip}/w}=0.35{\left(K_{o/w}\right)}^{0.81}$ (Eq.21)	7	1.43–5.49	Utilized the same dataset from Anderson et al. 1988 as described in Eq.20.	Experimental	Human	(Nitsche et al. 2006)
		$K_{\mathrm{sc},\mathrm{lip}/w}=0.43{\left(K_{o/w}\right)}^{0.81}$ (Eq.22)	72	−0.77–6.94	The authors pooled reported $K_{\mathrm{sc}/w}$ across multiple compounds and predicted corneocyte/water partition coefficients ( $K_{\mathrm{cor}/w}$ ) using a keratin-binding formula. $K_{\mathrm{sc},\mathrm{lip}/w}$ was subsequently estimated based on a ^a^two-phase model ( $K_{\mathrm{sc}/w}=\phi_{\mathrm{lip}}K_{\mathrm{sc},\mathrm{lip}/w}+\phi_{\mathrm{cor}}K_{\mathrm{cor}/w}$ ). This approach expanded the sample size of Eq.19 and recalibrated its coefficient from 0.35 to 0.43.	Experimental and derived	Human	(Nitsche et al. 2006)
		$K_{\mathrm{sc},\mathrm{lip}/w}=1.32{\left(K_{o/w}\right)}^{0.67}$ (Eq.23)	16	−0.13–5.49	Utilized the identical datasets from Anderson et al. 1988 and Johnson 1995 as described in Eq.20, combined with two additional datasets from Hansen et al. 2008 and Wang et al. 2010. In the two latter datasets, $K_{\mathrm{sc},\mathrm{lip}/w}$ was determined via partition equilibrium assays using either reconstituted human SC lipid membranes or extracted pure SC lipids.	Experimental	Human	(Hansen et al. 2012)
K_sc/ve_	SC/viable epidermis partition coefficients	^b^ $K_{\mathrm{sc}/\mathrm{ve}}=\frac{\left(1-f_{\mathrm{lip},\mathrm{sc}}\right)\;∙\;(f_{\mathrm{lip},\mathrm{sc}}\;∙\;K_{o/w})}{\left(1-f_{\mathrm{lip},\mathrm{ve}}\right)\;∙\;(f_{\mathrm{lip},\mathrm{ve}}\;∙\;K_{o/w})}$ $f_{\mathrm{lip},\mathrm{sc}}=0.05$ $f_{\mathrm{lip},\mathrm{ve}}=0.02$ (Eq.24)	−	−	Estimated based on the partitioning characteristics of compounds between lipid and aqueous phases; this formulation is also applicable for estimating dermis/blood partition coefficients ( $K_{\mathrm{dermis}/b}$ ).	Derived	−	(Shatkin and Brown 1991)
K_de/w_	Dermis/water partition coefficients	^c^ $K_{\mathrm{de}/w}=\frac{0.6}{f_{\mathrm{non}}}\;∙\;(0.68+\frac{0.32}{f_{u}}+0.001\;∙\;f_{\mathrm{non}}\;∙\;K_{o/w})$ (Eq.25)	22	−3.24–4.06	The authors pooled data from multiple studies, including Scheuplein and Blank 1973 and Cross et al. 2003. $K_{\mathrm{dermis}/w}$ was determined either experimentally via partition equilibrium assays using heat-separated dermis, or estimated indirectly based on the dermal water content (0.6). Additionally, $K_{\mathrm{dermis}/w}$ values for a subset of compounds were estimated via interpolation by the authors.	Experimental and derived	Human, guinea pig, rat and mouse	(Kretsos et al. 2007)
		^c^ $K_{\mathrm{de}/w}=0.7\;∙\;\left(0.68+\frac{0.32}{f_{u}}+0.025\;∙\;f_{\mathrm{non}}\;∙\;K_{\mathrm{sc},\mathrm{lip}/w}\right)$ (Eq.26)	−	−	Modified from Eq.25 by accounting for the actual dermal lipid content (2.5%) and substituting $K_{\mathrm{sc},\mathrm{lip}/w}$ for $K_{o/w}$ .	−	−	(Chen et al. 2014)
D_sc_ (cm^2^/h)	Diffusion coefficient for SC	^d^ $\frac{D_{\mathrm{sc}}}{l_{\mathrm{sc}}}=10^{\left(-2.8-0.006\;∙\;\mathrm{MW}\right)}$ (Eq.27)	93	18.1–764	Established by stripping the $K_{o/w}$ -dependent terms from Eq.17; the original dataset was derived from the IVPT dataset compiled by Flynn (1990).	Experimental and derived	Human	(Hoang 1992; Cleek and Bunge 1993)
D_sc,lip_ (cm^2^/h)	Diffusion coefficient for SC lipid	$D_{\mathrm{sc},\mathrm{lip}}=3600\;∙\;(1.24\;∙\;10^{-7})\cdot {\left(\frac{100}{\mathrm{MW}}\right)}^{2.43}+(2.34\;∙\;10^{-9})$ (Eq.28)	6	32.0–758	1. Hatcher and Plachy 1993: Direct determination of the diffusion coefficient for molecular oxygen via electron paramagnetic resonance (EPR) spin labeling in an unsaturated synthetic model lipid system. 2. Johnson et al. 1996: Video-fluorescence recovery after photobleaching (VFRAP) measurements of lateral diffusivities of fluorescent probes within bilayers reconstituted from extracted SC lipids.	Experimental	Human and Synthetic	(Wang et al. 2007)
		^e^ $D_{\mathrm{sc},\mathrm{lip}}=\;\left\{ \begin{array}{l} 2\;∙\;10^{-5}\;∙\;e^{-0.46\;∙\;r^{2}},\mathrm{MW}\leq 380\\ 3\;∙\;10^{-9},\mathrm{MW}>380 \end{array}\right.$ (Eq.29)	−	−	First-principles theoretical derivation based on scaled particle theory and Wilke-Chang correlation	Derived	−	(Mitragotri 2003)
D_de_ (cm^2^/h)	Diffusion coefficient for dermis	^c^ $D_{\mathrm{de}}=\frac{3600\;∙\;10^{-4.15-0.655∙\;\log\;\mathrm{MW}}}{\left(0.68+\frac{0.32}{f_{u}}+0.001\;∙\;f_{\mathrm{non}}\;∙\;K_{o/w}\right)}$ (Eq.30)	21	18–477	$D_{\mathrm{dermis}}$ was calculated using the equation $k_{p}=D\cdot K/h$ , where the experimental $k_{p}$ values were collected from multiple IVPT studies, and the corresponding $K_{\mathrm{dermis}/w}$ values were determined using the methodology described in Eq. 25.	Experimental and derived	Human, guinea pig, rat and mouse	(Kretsos et al. 2007)
		^c^ $D_{\mathrm{de}}=\frac{3600\;∙\;10^{-4.15-0.655\;∙\;\log\;\mathrm{MW}}}{\left(0.68+\frac{0.32}{f_{u}}+0.025\;∙\;f_{\mathrm{non}}\;∙\;K_{\mathrm{sc},\mathrm{lip}/w}\right)}$ (Eq.31)	−	−	Modified from Eq. 28 by accounting for the actual dermal lipid content (2.5%) and substituting $K_{\mathrm{sc},\mathrm{lip}/w}$ for $K_{o/w}$ .	−	−	(Chen et al. 2014)
		^c^ $D_{\mathrm{de}}=\frac{3600\;∙\;10^{-4.38-0.207∙{\mathrm{MW}}^{\frac{1}{3}}}}{\left(0.68+\frac{0.32}{f_{u}}+0.025\;∙\;f_{\mathrm{non}}\;∙\;K_{\mathrm{sc},\mathrm{lip}/w}\right)}$ (Eq.32)	−	−	This is an adaptation based on Eq.30 reported by Kretsos et al. (2007) and Eq.30 reported by Chen et al. (2014).	−	−	(Patel et al. 2022)

Note: - Indicates that the equation was derived theoretically or modified from previously reported models without experimental data fitting.

^a^ In the two-phase model, $\phi_{\mathrm{lip}}$ and $\phi_{\mathrm{cor}}$ denote the volume fractions of the lipid and corneocyte phases, respectively, and $K_{\mathrm{cor}/w}$ denotes the SC corneocyte/water partition coefficient.

^b^ $f_{\mathrm{lip},\mathrm{sc}}$ and $f_{\mathrm{lip},\mathrm{ve}}$ represent the lipid fractions in the SC and VE, respectively.

^c^ $f_{u}$ is fraction unbound in a 2.7% albumin solution at pH 7.4, and $f_{\mathrm{non}}$ is the fraction of non-ionized solute at pH 7.4.

^d^ $l_{\mathrm{sc}}$ denote the apparent thickness of SC (cm).

^e^ r represents the molecular radius in Angstroms ( Å ).

## 5. Comparison of hypotheses and architectures for current skin PBPK model

Currently, transdermal PBPK models are primarily developed using well-established and validated modeling platforms such as Simcyp, GastroPlus, Skin-CAD, and PK-Sim/MoBi. These models differ in their consideration of physiological factors, structural assumptions, and TDS formulation handling (Table 3), offering specific advantages depending on the drug type and clinical scenario.

**Table 3.** Comparison of model structural hypotheses across different skin PBPK software.

Model hypothesis	Simcyp (Patel 2015; Patel et al. 2022)	GastroPlus (Spires 2020; van Osdol et al. 2024)	Skin-CAD (Mori and Tojo 2006)	PK-Sim/MoBi (Hamadeh and Sevestre 2013)
**Physiological factors**				
Anatomical Sites	Incorporates effects of anatomical site on SC, VE, and dermis thickness; SC pH and hydration; blood flow; and hair follicle density.	Incorporates effects of anatomical site on SC, VE, and dermis thickness; blood flow; and hair follicle density.	Not predefined, manual adjustment of skin thickness is available.	Not predefined, manual adjustment of skin thickness is available.
Sex	Male forehead, forearm, and face pH is lower than that of females. Male SC lipid content is lower than that of females.	−	−	−
Age	−	Incorporates effects of age on skin thickness in pediatrics.	−	Incorporates effects of age on skin thickness, blood flow, and hydration in pediatrics.
**Skin structure**				
Stratum corneum	Mechanistic ‘bricks-and-mortar’ corneum<piologic ‘brick-and-mortar’ structure; discretized into ≥12 layers depending on the anatomical site, with the forehead exhibiting the fewest layers at 12. Accounts for depth-dependent increases in corneocyte hydration across SC, which result in increased corneocyte thickness and volume. Mechanistically incorporates keratin binding of non-ionized drug and ionization-based entrapment within corneocytes.	Homogeneous compartment; discretized into 10 layers; considers keratin binding.	Homogeneous compartment; discretized into multiple grids; considers keratin binding.	Homogeneous compartment; discretized into 40 layers.
Viable epidermis	Homogeneous compartment modeled as a single non-discretized layer; considers metabolism and tissue binding.	Homogeneous compartment; discretized into 5 layers; considers metabolism and tissue binding.	VE and dermis as a single homogeneous compartment; discretized into multiple grids; considers metabolism and tissue binding.	Homogeneous compartment; discretized into 40 layers; incorporates metabolism.
Dermis	Homogeneous compartment modeled as a non-discretized single layer; considers metabolism, tissue binding, and systemic circulation uptake.	Homogeneous compartment; discretized into 5 layers; incorporates systemic circulation uptake.	Incorporated into VE; systemic circulation uptake at the dermis boundary.	Homogeneous compartment; discretized into 40 layers; incorporates metabolism.
Appendages	Shunt transport; drug enters dermis by bypassing the SC.	Shunt transport; drug enters VE and dermis bypassing the SC.	−	Shunt transport; drug enters dermis by bypassing the SC.
**Formulation factors**				
Applicable formulations	Solutions, suspensions, gels, emulsions, ointments, patches, etc.	Solutions, suspensions, gels, emulsions, creams, ointments, patches, etc.	Solutions, patches, semi-solid formulations.	Solutions, suspensions, gels, emulsions, creams, ointments, etc.
Formulation systems	Three-phase system.	Three-phase system.	Two-phase system.	Two-phase system.
Dynamic processes	Continuous phase drug absorption; transfer between dispersed and continuous phases; solid particle dissolution; excipient evaporation; drug precipitation.	Continuous and dispersed phase drug absorption; transfer between dispersed and continuous phases; solid particle dissolution; excipient and drug evaporation; drug precipitation.	Continuous phase drug absorption and solid particle dissolution.	Continuous phase drug absorption and drug evaporation.
Special formulations	−	−	Enhancement of drug absorption via microneedles and iontophoresis.	−

Note: - indicates that the factor is not predefined or quantified by the software.

Regarding the physiological factors, Simcyp uses a comprehensive database to highlight how sex affects skin pH and SC lipid content, while also incorporating site-specific skin pH, hydration, thickness, blood flow, and hair follicle density. In contrast, GastroPlus and PK-Sim/MoBi focus on age-related factors, integrating developmental characteristics such as pediatric skin thickness and blood flow. Although Skin-CAD lacks preset physiological databases, it allows users to manually adjust skin thickness.

For skin physiology, Simcyp, GastroPlus, and PK-Sim/MoBi employ multi-layer skin models consisting of SC, VE, and dermis. Conversely, Skin-CAD simplifies the VE and dermis into a unified compartment. Building upon these multi-layer structures, Simcyp, GastroPlus, and PK-Sim/MoBi incorporate appendageal absorption pathways (Figure 4c). Notably, GastroPlus uniquely characterizes lateral drug diffusion between appendageal sebum and surrounding tissues, extending beyond simple longitudinal permeation. In terms of microstructure, Simcyp utilizes a unique ‘bricks-and-mortar’ structure and depth-dependent hydration profiles to simulate the heterogeneity of the SC, whereas the other three platforms assume the SC to be a homogeneous compartment. The ‘bricks-and-mortar’ structure in Simcyp assumes that interlayer drug diffusion occurs exclusively within the intercellular lipid ‘mortar’ (Figure 4a) (characterized by the diffusion coefficient, D_sc,lip_). Simultaneously, within each sub-layer, the drug can partition between the lipid and the corneocytes phases based on the corneocyte permeability (P_cell_). Because the model prohibits direct transcellular permeation (Figure 4b) into subsequent layers, the drug must bypass the corneocytes, thereby creating a highly tortuous diffusion pathway. Consequently, the effective diffusion path length (L_eff_) is defined as the product of corneocyte thickness (H_corn_) and lipid tortuosity (Tort). Within the modeling architecture, H_corn_ is a calculated parameter assigned per individual SC layer. Because drug must navigate around each distinct cell layer along the intercellular pathway, the resulting total L_eff_ is substantially greater than the absolute physical thickness of the SC. Furthermore, Simcyp incorporates a depth-dependent hydration gradient across the SC, as depth increases across individual layers, elevated hydration levels lead to a corresponding increase in H_corn_. This spatial heterogeneity mechanistically replicates the nonlinear permeation dynamics of drug within the SC. In contrast, the homogeneous compartment approach assumes a uniform diffusion environment across all SC sub-layers. Such models employ an apparent diffusion coefficient (D_sc_) that lumps the lipid and corneocyte diffusion pathway, thereby neglecting the spatial heterogeneity of the SC. In addition, all four software platforms account for drug metabolism in the viable skin layers, Simcyp, GastroPlus, and Skin-CAD incorporate keratin binding within the SC, whereas PK-Sim/MoBi omit keratin binding (Table 3). Consistent with Section 4.3.1, Simcyp, GastroPlus, and PK-Sim/MoBi solve the governing PDEs using the FDM method. To balance accuracy and efficiency, each platform uses a different spatial resolution: PK-Sim/MoBi finely discretizes the SC into 40 layers, offering significantly higher spatial resolution than Simcyp (≥12 layers depending on the anatomical site) and GastroPlus (10 layers). Additionally, Skin-CAD employs a multi-grid approach based on Fick's second law.

The kinetic processes of the formulation system are critical determinants of the percutaneous absorption rate. Both Simcyp and GastroPlus use complex triphasic system models to simulate the release behavior of various formulations, including solutions, suspensions, and emulsions. Notably, GastroPlus allows drug absorption from both continuous and dispersed phases, whereas Simcyp primarily focuses on absorption from the continuous phase. Regarding formulation dynamic changes, both Simcyp and GastroPlus integrate mechanisms for active ingredient precipitation driven by solvent evaporation, though GastroPlus also accounts for the evaporative loss of the volatile drug itself. In comparison, Skin-CAD and PK-Sim/MoBi utilize simplified biphasic models, with Skin-CAD relies on a classical diffusion model to characterize drug release. Additionally, Skin-CAD provides specialized modules for microneedles and iontophoresis to characterize the absorption of enhanced delivery systems.

## 6. Comparison of application cases for current skin PBPK model

We reviewed skin PBPK research published through January 2026, identified through a literature search of the PubMed database using keywords such as ‘dermal PBPK’ and ‘specific software names’. We summarized the findings in Table 4 to evaluate the application potential, reliability, and versatility of these models. The results indicate that skin PBPK models have been integrated into multiple stages of TDS product development, providing robust quantitative support across various clinical scenarios. These primary applications include predicting local and systemic PK and PD following TDS administration, forecasting PK in special populations, assessing how formulation factors affect absorption, and evaluating safety following dermal chemical exposure. Overall, skin PBPK research facilitates the assessment of how physiological variations like anatomical site, age, and sex influence PK. Furthermore, it supports virtual bioequivalence (VBE) trials for generic drug development and facilitates formulation optimization, thereby accelerating market entry and reducing research and development costs.

**Table 4.** Research case studies of skin PBPK models.

Software	No.	Drug	Formulation	Subjects	Administration site	Objective	Conclusion	Application scenarios	Source
Simcyp	1	Diclofenac	Spray, gel, patch, lotion	Caucasian and Japanese healthy volunteers; patients with knee joint effusion	Forearm, thigh, back, and knee	To develop a mechanistic skin PBPK model for predicting PK following transdermal administration under different physiological states (e.g. sex, race, and site differences).	The model effectively characterized PK following transdermal administration across different populations and formulation types.	PK prediction: diverse formulations and patient populations	Polak et al. (2012))
	2	Ketoprofen	Solution, Gel, Patch	Healthy volunteers	Arm, Back, Knee	To explore the model's predictive performance for local (muscle) and systemic exposure following transdermal administration of ketoprofen.	The model successfully characterized the local and systemic PK of topical ketoprofen formulations.	PK prediction: diverse formulations	Polak and Patel (2018)
	3	Clindamycin	Solution and Gel	Healthy volunteers	Not specified	To apply the model to explore the impact of formulation differences on the PK of clindamycin after transdermal administration.	The model distinguished and quantified the impact of formulation differences on PK, aiding in formulation optimization.	PK prediction: diverse formulations	Polak et al. (2018)
	4	Lidocaine	Patch and cream	Healthy, Geriatric, and Pediatric subjects	Not specified	To explore the model's PK predictive performance in healthy, geriatric, and pediatric populations using various lidocaine formulations.	The model reasonably predicted and quantified the impact of age on the transdermal absorption of lidocaine.	PK prediction: diverse formulations; special populations (geriatric and pediatric)	Salem et al. (2019)
	5	Selegiline	Patch	Healthy volunteers and geriatric subjects	Upper arm	To predict the PK of selegiline in special populations and identify influential factors.	Compared to healthy adults, exposure remained unchanged in adolescents but increased in patients with renal or hepatic impairment.	PK prediction: special populations (geriatric)	Puttrevu and Arora 2020)
	6	Timolol	Patch	Healthy volunteers	Upper arm	To validate the model's predictive performance for the PK of timolol after transdermal administration.	The model effectively characterized the PK of the timolol patch.	PK prediction	Patel et al. (2015)
	7	Nicotine	Patch	Healthy volunteers	Upper arm	To predict and validate the PK and PD of nicotine following transdermal administration.	The model effectively predicted nicotine PK and, linked with PD, predicted cardiovascular efficacy.	PK/PD prediction	Martins et al. (2018)
	8	Nimesulide	Gel	Healthy volunteers	Not specified	To characterize the *in vitro* and *in vivo* absorption profiles of nimesulide gel to support formulation optimization and clinical development.	Successfully predicted *in vitro* and *in vivo* PK, supporting the model's application in virtual bioequivalence (VBE) trials.	PK prediction	Mittapelly and Polak (2022)
	9	Caffeine	Solution	Healthy volunteers	Chest	To quantitatively analyze the contribution of the hair follicle pathway to the transdermal absorption of caffeine.	Hair follicles contributed 27% to caffeine absorption.	PK prediction: assessment of hair follicle absorption pathway	Martins et al. (2017)
	10	Trifarotene	Cream	Healthy volunteers	Not specified	To predict local and systemic PK, as well as CYP-mediated DDI, following transdermal trifarotene administration.	The model effectively predicted PK and confirmed a very low risk of CYP-mediated DDI.	PK prediction: DDI assessment	Patel et al. (2017)
	11	Finasteride and Minoxidil	Combined solutions	Healthy volunteers and male alopecia patients	Scalp	To explore the PK predictive performance of the PBPK model following scalp administration, which involves significant physiological differences.	The model successfully predicted the PK and PD of the study drugs after scalp administration.	PK prediction: scalp administration	Ngampanya et al. (2021)
	12	Bisphenol A	Thermal paper	Healthy volunteers	Palm and front surface of fingers	To predict and validate the PK of Bisphenol A following transdermal absorption.	Successfully predicted local and systemic PK and explained the reservoir effect of the SC.	PK prediction: toxicity assessment	Wiśniowska et al. (2023)
	13	Diclofenac	Gel	Healthy volunteers	Not specified	To predict local and systemic exposure of generic and brand-name diclofenac to waive clinical endpoint bioequivalence studies.	Supported regulatory decision-making by waiving certain generic drug consistency evaluation studies.	VBE	Tsakalozou et al. (2021)
	14	Desoximetasone	Spray	Healthy volunteers and simulated patients with skin disease	Abdomen	To clarify the impact of formulation factors (e.g. solubility, viscosity) and physiological differences on bioequivalence assessment.	Drug solubility in the formulation is a key factor affecting PK; skin lesions reduce the influence of the formulation.	VBE	Clarke et al. (2022)
	15	Metronidazole	Solution, Gel, and Creams	Healthy volunteers	Volar forearm	To explore a workflow combining PBPK models and *in vitro* IVPT data to predict local SC exposure after metronidazole administration.	Utilizing *in vitro* data, the model effectively predicted local exposure through IVIVE.	IVIVE	Arora et al. (2022)
	16	Caffeine, Crisaborole, Ketoprofen	Caffeine solution, Crisaborole ointment, Ketoprofen solution	Healthy volunteers	Not specified	To improve the predictive performance of the skin PBPK model using absorption parameters optimized from porcine skin IVPT data.	Porcine ear IVPT effectively improved PK prediction accuracy compared to QSAR models.	IVIVE	Krumpholz et al. (2025)
	17	Caffeine	Solution	Porcine *in vitro* skin	Porcine ear	To develop a porcine ear PBPK model for predicting drug permeation behavior through porcine skin *in vitro*.	The model effectively distinguished differences in caffeine permeation between *in vitro* porcine ear and human skin.	*In vitro* IVPT prediction	Krumpholz et al. (2024a)
	18	Caffeine	Solution	Excised human skin	Abdomen, breast, and thigh	To evaluate the model's accuracy in predicting *in vitro* human skin IVPT absorption data using caffeine as a model drug.	Due to data heterogeneity, the model struggled to effectively predict human skin IVPT data across all laboratories.	*In vitro* IVPT prediction	Krumpholz et al. (2024b)
GastroPlus	1	Diclofenac, Salicylic acid, Coumarin, Nicotine, Caffeine	Gel, Solution, Cream, Patch	Healthy volunteers	Back, Chest, Face and neck	Evaluate the PK prediction performance of the model for TDS administration.	The model can effectively predict PK based on *in vitro* data and IVIVE.	PK prediction	Li et al. (2022)
	2	Clobetasol propionate	Cream	Psoriasis patients	Arm non-lesional skin	Predict the local dermal PK of Clobetasol propionate and evaluate the impact of formulation properties on local exposure.	The model reasonably predicts local exposure, and sensitivity analysis identifies the impact of drug solubility on local exposure	PK prediction: local	van Osdol et al. (2024)
	3	Phenoxyethanol (PhE) and its metabolite Phenoxyacetic acid (PhAA)	Solution and cream	Rat and healthy volunteers	Not specified	Predict the tissue and urinary exposure in rats and humans after skin contact with PhE and PhAA, to assess the safety risks of cosmetics.	The model accurately predicts the PK of PhE and PhAA, determining their safe dose range.	PK prediction: toxicity assessment	Zhang et al. (2022)
	4	Methylenediphenyl diisocyanate (MDI) substances	Solution	Virtual healthy volunteers	Not specified	Predict the absorption of MDI components (39 types) after skin contact to support safety evaluation.	MDI monomers have high percutaneous absorption but low absorption rates, with absorption decreasing as molecular weight and carrier lipophilicity increase	PK prediction: toxicity assessment	Bartels et al. (2022)
	5	Oxybenzone	Lotion, Spray	Healthy volunteers	Abdomen	Establish a model using *in vitro* data to predict systemic exposure of Oxybenzone in different sunscreen formulations.	The model effectively predicts the PK of Oxybenzone by integrating formulation-specific *in vitro* absorption data.	PK prediction: toxicity assessment	Golbamaki et al. 2025)
Skin-CAD	1	A novel compound: GTS-21	Patch	Hairless rats and hairless Rat Skin	Abdominal	Use *in vitro* IVPT data to assess the model's prediction performance of *in vivo* exposure after GTS-21 patch administration.	The model effectively predicts *in vivo* PK of GTS-21 in hairless rats.	IVIVE	Kawamata and Tojo (2002)
	2	*β*-Estradiol, progesterone, verapamil, etc. (19 drugs with varying molecular weights and lipophilicity)	Solution (*in vitro*), Patch (*in vivo*)	Healthy volunteers	Not specified	Explore the relationship between 3D human skin culture model (LabCyte) and *in vitro* absorption parameters of hairless mouse skin, and predict PK after transdermal absorption.	Successfully established correlation between LabCyte and hairless mouse skin absorption, and effectively predicted human PK after transdermal absorption of different drugs via IVIVE.	IVIVE	Hikima et al. (2012)
	3	Tulobuterol	Patch	Healthy volunteers	Not specified	Evaluate the model's ability to predict tulobuterol patch PK by combining *in vitro* release and permeation results of hairless mouse.	The model can effectively predict PK based on the *in vitro* data, providing an effective method for evaluating the clinical performance of transdermal TDS.	IVIVE	Nakamura et al. (2012)
	4	Felbinac, Ketoprofen, Flurbiprofen, Loxoprofen	Patch	*In vitro* Hairless mouse skin, virtual healthy volunteers	Abdominal	Use *in vitro* IVPT data to explore the effect of environmental temperature on the absorption of NSAID patches.	The model shows that increased environmental temperature enhances drug absorption by increasing drug solubility in the SC.	IVIVE: assessment of the impact of temperature	Tominaga and Tojo (2010)
	5	Nicotine and Isosorbide dinitrate (ISDN)	Patch	Healthy volunteers and *in vitro* human skin	Abdomen and Back	Explore the use of varying humidity in IVPT to predict *in vivo* PK.	For accurate *in vivo* PK prediction, IVPT experiments should be conducted at clinically relevant humidity (about 50%).	IVIVE: assessment of the impact of humidity	Ishida et al. (2015)
	6	Prednisolone	Patch	Rats	Not specified	Develop a new Prednisolone patch formulation and evaluate its clinical application advantages over oral and topical eye drops in maintaining *in vivo* exposure.	Compared to oral administration, Prednisolone patches provide more stable and effective systemic exposure; compared to topical eye drops, they provide higher ocular exposure.	PK prediction	Isowaki et al. (2003)
	7	Fentanyl	patch	Healthy volunteers, Patients (PD)	Not specified	Evaluate the model's prediction performance of fentanyl patch PKPD after administration, and optimize clinical study design.	By combining *in vitro* release, IVPT data, and PKPD relationships, the model effectively predicts the PKPD characteristics of Fentanyl patches.	PK/PD prediction	Mori and Tojo (2006)
	8	Verapamil	Microneedles, patch	Virtual healthy volunteers	Not specified	Evaluate the impact of skin metabolism on drug absorption and systemic PK after microneedle administration, and compare it with traditional patches.	Compared to patches, microneedles absorb faster, and skin metabolism has negligible effects on the PK after transdermal administration.	PK prediction: Microneedle	Al-Qallaf et al. 2009)
	9	Fentanyl	Microneedles	Virtual human skin digital model	Arm	Quantitatively explore the relationship between microneedle geometry and its effect on skin compression strain and skin diffusion coefficient.	Skin compression strain reduces drug absorption, and increasing microneedle density and size increases this reduction effect	PK prediction: Microneedle	Olatunji et al. (2011)
	10	Fentanyl	Microneedles	Virtual healthy volunteers	Not specified	Evaluate the impact of microneedle geometry on fentanyl PK after transdermal administration	Increasing microneedle length and array density significantly enhances Fentanyl transdermal permeability and steady-state PK	Microneedle formulation optimization	Al-Qallaf et al. (2007)
	11	Insulin	Microneedles	Virtual healthy volunteers	Not specified	Quantitatively analyze the impact of microneedle geometry on transdermal absorption parameters and systemic drug concentrations.	The model identifies the optimal microneedle radius and penetration depth to achieve target exposure.	Microneedle formulation optimization	Olatunji et al. (2009)
	12	High molecular weight drugs: Calcein, Insulin, Bovine Serum Albumin (BSA), Nanosphere particles	Microneedles	Virtual healthy volunteers	Abdomen, Back, leg, thigh, Chest, Sole	Explore the interaction between microneedle geometry and skin physiological characteristics on drug transdermal permeation.	The results suggest that microneedle geometry design must consider skin physiological differences.	Microneedle formulation optimization	Al-Qallaf and Das (2009)
	13	Tulobuterol	patch	*In vitro* hairless mouse skin and virtual healthy volunteers	Simulated SC thicknesses to represent different sites	Predict the bioequivalence of tulobuterol patch (innovator vs. generic) after administration to different skin sites.	The bioequivalence of Tulobuterol is affected by SC thickness.	VBE	Tojo and Hikima (2007)
	14	Ketotifen	Paste	*In vitro* hairless mouse skin and *in vivo* rabbits	Eyelid	Develop a novel ketotifen paste formulation to improve absorption bioavailability and drug effect duration.	*In vitro* skin IVPT data effectively predict the *in vivo* PK of ketotifen paste, maintaining stable and effective drug exposure.	New formulation development	Kimura and Tojo (2007)
PK-Sim/MoBi	1	Theophylline, Salicylic acid, Hydrocortisone	Solution	Children and Adults	Volar forearm	Develop a skin PBPK model suitable for children (from neonates to adults) based on skin physiology and assess prediction performance.	Age-dependent skin physiology significantly affects drug absorption, and the model successfully predicts absorption characteristics in different age groups.	PK prediction: special populations (pediatric)	Yun et al. 2022)
	2	Loratadine, Chlorpheniramine maleate, Itraconazole	Microneedles	Landrace pigs and *in vitro* Porcine ear skin	Porcine ear skin	Integrate microneedle geometry and *in vitro* release to accurately predict local and systemic exposure after microneedle administration.	The model effectively predicts local and systemic PK of various drugs, confirming the advantages of the model in optimizing microneedle formulation for preclinical development.	PK prediction: microneedle	Railic (2025)

Due to the distinct underlying modeling hypotheses, the application focus of these four software platforms varies significantly (Table 4). Simcyp is the most active and widely utilized platform in clinical evaluation; it excels in PK predictions for special populations (e.g. pediatric and geriatric cohorts), drug‒drug interaction (DDI) assessments, and complex local-to-systemic exposure correlations (e.g. cases involving diclofenac and ketoprofen). GastroPlus, leveraging its sophisticated formulation modules, demonstrates high reliability in differentiating between dosage forms (solutions, gels, and patches) and in toxicological assessments for chemicals, such as determining safe dosage ranges for cosmetics. Skin-CAD exhibits specialized expertise in *in vitro-to-in vivo* extrapolation (IVIVE) and the field of drug-device combinations, such as microneedle-based delivery. Finally, the strengths of PK-Sim/MoBi lie in its open-source nature and the mechanistic exploration of pediatric skin models, but currently, limited research cases are available for it to fully demonstrate its broad reliability and versatility in diverse applications.

## 7. Limitations of current skin PBPK research

### 7.1. Complexity and inconsistency of model structures

Despite significant progress, current skin PBPK models face common limitations. First, existing software platforms employ varying structural assumptions and levels of complexity. This diversity creates methodological dilemmas for researchers attempting to code and construct independent skin PBPK models. For instance, is it truly indispensable to incorporate a heterogeneous ‘bricks-and-mortar’ structure for the SC, as implemented in Simcyp? How should the optimal degree of spatial discretization for different skin layers be determined? Furthermore, is the inclusion of appendageal pathways essential?

Regarding the selection between heterogeneous and homogeneous SC structural models, both approaches can effectively predict local tissue PK and systemic exposure, provided that accurate absorption parameters are obtained. However, both model types exhibit distinct advantages and limitations. Simplified homogeneous models require fewer parameters to effectively predict drug absorption. Concurrently, this minimal parameterization demands higher parameter accuracy, making these models inherently more reliant on high-quality IVPT data. Conversely, heterogeneous models feature a ‘bricks-and-mortar’ structure meticulously characterizing skin anatomy. This allows them to mechanistically predict absorption parameters based on QSAR models, reduce reliance on IVPT data, and simulate local exposure under altered physiological states, such as varied hydration levels (Chen et al. 2008; Patel et al. 2022). However, the introduction of numerous micro-scale parameters that are exceedingly difficult to measure experimentally or via QSAR model prediction (e.g. P_cell_) in heterogeneous models inherently increases the uncertainty of model predictions. Furthermore, comparative analyzes of both models using identical experimental data have demonstrated their equivalence in characterizing the spatiotemporal distribution dynamics within the SC for molecules of varying polarities, such as 4-cyanophenol, flufenamic acid, and caffeine (Stinchcomb et al. 1999; Chen et al. 2008; Hansen et al. 2008; Naegel et al. 2008). Given that the primary objective of skin PBPK modeling is to predict local tissue exposure and systemic circulation rather than specific corneocyte distribution, selecting a homogeneous model serves as an alternative approach that maximizes computational efficiency and significantly mitigates parameter identifiability challenges when robust IVPT data are available.

While a finer degree of spatial discretization theoretically yields higher predictive accuracy, the corresponding increase in computational burden must be considered. In practice, modelers can evaluate the relationship between varying degrees of discretization and predictive performance for IVPT absorption profiles. This evaluation identifies the minimum level of discretization that accurately predicts the IVPT curve before linking the dermal component to systemic circulation.

Regarding appendageal pathways, a homogeneous structure omitting these routes is generally sufficient for application sites lacking a high density of hair follicles. The contribution of the appendageal route to overall percutaneous absorption depends on the interplay between the application site and specific drug properties. While specialized anatomical areas with high hair follicle density (such as the scalp or face) often necessitate the inclusion of appendageal pathways, simplified homogeneous models remain highly robust for general application sites.

### 7.2. Challenges in accurate parameterization

The second limitation is that inaccurate parameter sources introduce prediction bias. Obtaining diffusion and partition coefficients for distinct skin layers from human skin experimental data remains highly challenging. Consequently, current QSAR models serving as alternatives to predict absorption parameter.

However, as summarized in the QSAR model establishment methods in Table 2, the accuracy and generalizability of QSAR models are inherently constrained by their underlying experimental training databases, which often feature small sample sizes and narrow physicochemical property ranges. Another major limitation of QSAR models is their inability to effectively predict how different vehicles influence the diffusion and partitioning capabilities of the same compound (Kasting 2013). These limitations apply to QSAR models predicting apparent diffusion and partition coefficients (D_sc_, K_sc/w_) based on IVPT data. They also apply to models predicting microscale parameters, such as D_sc,lip_ and K_sc,lip/w_, derived from experiments that isolate SC lipids structures. These limitations inevitably result in significant prediction bias during PBPK modeling and simulation.

**Figure 8.**
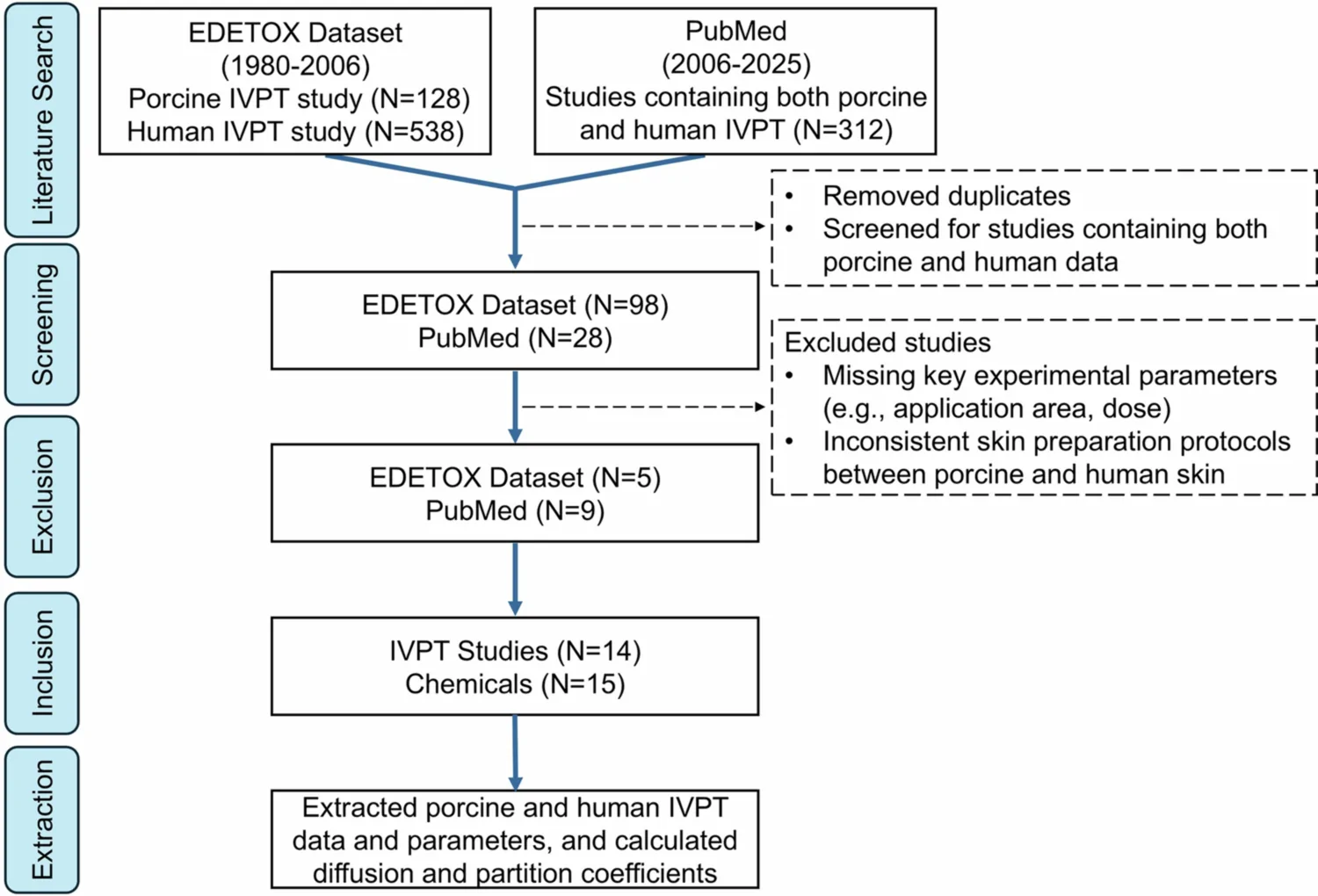
IVPT studies screening strategy.

**Figure 9.**
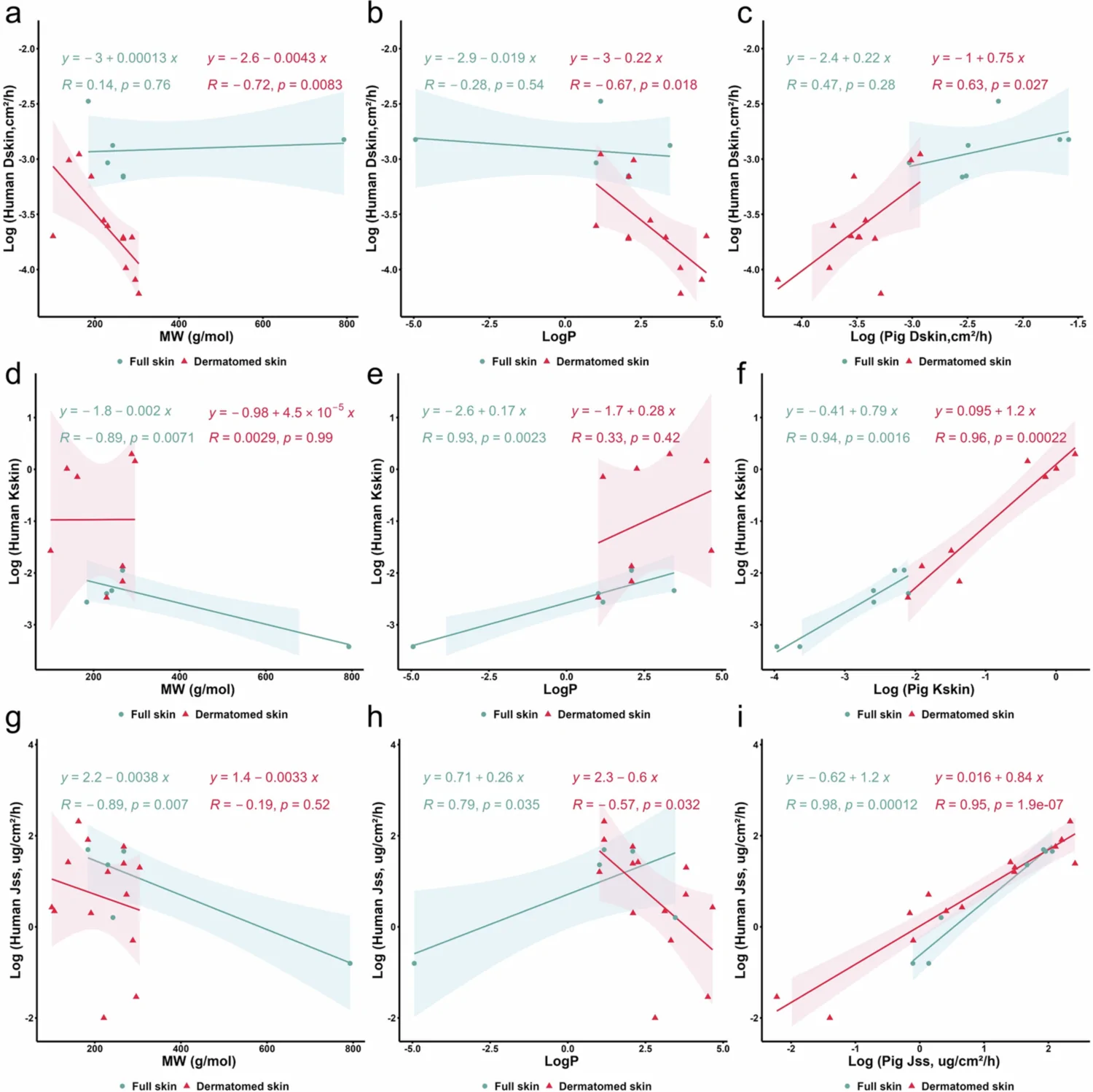
Correlations of human skin diffusion coefficients (D_skin_), partition coefficients (K_skin_) and steady-state flux (J_ss_) with drug physicochemical properties (MW (a, d, g) and LogP (b, e, h)) and with corresponding pig skin parameters (c, f, i). Red triangles and green circles represent the measured values of D_skin_, K_skin_, or J_ss_ for dermatomed skin and intact skin, respectively. The solid lines represent general linear regression lines; the linear relationship was evaluated using Pearson correlation analysis, with significance tested via t-test.

To reduce QSAR prediction bias, porcine skin IVPT data have been proposed as a surrogate for human IVPT, but some prediction bias persists due to interspecies differences (Krumpholz et al. 2025). Therefore, this study systematically searched recent literature (2006 to 2025) and the EDETOX database (1980 to 2006, Evaluations and Predictions of Dermal Absorption of Toxic Chemicals) (EDETOX 2005) to extract paired human and porcine IVPT experimental data. The IVPT data screening strategy and compiled results are provided in Figure 8 and Table 5. Subsequently, a correlation analysis was performed to evaluate the relationships of physicochemical and porcine IVPT parameters with human IVPT parameters (including J_ss_, and D and K derived from Equations 14–15). The results are presented in Figure 9. This exploratory analysis aimed to evaluate the potential of using porcine IVPT data as a surrogate for human skin absorption parameters compared to physicochemical property-based predictions. The analysis results reveal that physicochemical properties are not as strongly correlated with key model absorption parameters (Figure 9a–b, d–e, and g–h), which indirectly highlights the intrinsic prediction bias of QSAR models. Consistent with this analysis, Korinth et al. evaluated the experimental percutaneous penetration parameters of 11 compounds and compared them with predictions from multiple QSAR models, revealing substantial discrepancies between the predicted and experimental values (Korinth et al. 2012). Encouragingly, as shown in Figure 9c, f, and i, human IVPT parameters demonstrate a stronger correlation with pig IVPT parameters than with physicochemical properties, although they are not perfectly equivalent.

**Table 5.** Literature sources and extracted parameters from the included IVPT studies.

Chemical	Vehicle	Skin		J_max_ (ug/cm2/h)		t_lag_ (h)		Reference
		Porcine	Human	Porcine	Human	Porcine	Human	
Nicotine	Phosphate Buffer	Dermatomed (0.765 mm)	Dermatomed (0.408 mm)	218	206	0.825	0.883	Qvist et al. (2000)
Salicylic acid	Phosphate Buffer	Dermatomed (0.765 mm)	Dermatomed (0.408 mm)	25.7	26.2	1.00	1.00	Qvist et al. (2000)
Testosterone	Phosphate Buffer, 20% ethanol	Dermatomed (0.765 mm)	Dermatomed (0.408 mm)	0.792	0.501	3.00	5.00	Qvist et al. (2000)
Diazinon	Acetone	Dermatomed (0.5 mm)	Dermatomed (0.5 mm)	30.0	20.0	0.800	6.90	Moody and Nadeau (1994)
N,N-Diethyl-m-toluamide	Acetone	Dermatomed (0.5 mm)	Dermatomed (0.5 mm)	0.700	2.00	1.40	0.600	Moody and Nadeau (1993)
Brilliant Blue	40% Propylene Glycol and 10% N-Methyl-2-pyrrolidone	Full Thickness	Full Thickness	0.783	0.158	0.208	3.59	Príborský and Mühlbachová (1990)
2,4-Dichlorophenoxyacetic acid	Acetone	Dermatomed (0.5 mm)	Dermatomed (0.5 mm)	0.04	0.01	1.1	1.50	Moody et al. (1994)
Diisopropyl-fluorophosphate1	Neat	Full-thickness (1.90 ± 0.41 mm)	Full-thickness (1.90 ± 0.41 mm)	84.0	49.5	1.00	1.80	Vallet et al. (2007)
Demeton-S-methyl	Neat	Full-thickness (1.90 ± 0.41 mm)	Full-thickness (1.90 ± 0.41 mm)	46.5	23.0	6.40	6.50	Vallet et al. (2007)
Demeton-S-methyl	Neat	Split-thickness (0.61 ± 0.14 mm)	Dermatomed (0.61 ± 0.14 mm)	29.5	16.0	3.20	2.50	Vallet et al. (2007)
O-ethyl-S-[2(di-isopropylamino)ethyl] methyl phosphonothioate (VX)	Neat	Full-thickness (1.72 ± 0.08 mm)	Full-thickness (1.72 ± 0.08 mm)	90.5	45.5	1.60	7.00	Vallet et al. (2008)
O-ethyl-S-[2(di-isopropylamino)ethyl] methyl phosphonothioate (VX)	Neat	Split-thickness (0.44 ± 0.01 mm)	Dermatomed (0.44 ± 0.01 mm)	129	57.5	0.700	1.70	Vallet et al. (2008)
O-ethyl-S-[2(di-isopropylamino)ethyl] methyl phosphonothioate (VX)	Neat	Split-thickness (530 ± 50 μm)	Dermatomed (430 ± 30 μm)	260	24.5	1.40	2.40	Millerioux et al. (2009)
O-ethyl-S-[2(di-isopropylamino)ethyl] methyl phosphonothioate (VX)	Neat	Full-thickness (1.7 ± 0.1 mm)	Full-thickness (1.7 ± 0.1 mm)	115	45.5	1.70	7.00	Rolland et al. (2011)
heptane	JP-8 jet fuel	Dermatomed (500 μm)	Dermatomed (500 μm)	4.54	2.67	1.49	2.08	Singh et al. (2002)
Benzoyl Peroxide	Oil-based matrix	Full-thickness	Full-thickness	2.15	1.60	1.88	4.52	Brighenti et al. (2025)
Carprofen	Phosphate Buffered Saline	Dermatomed (700 μm)	Dermatomed (700 μm)	1.37	5.10	4.58	7.92	Parra et al. (2016)
Diclofenac	10 mmol/L MES/HBSS buffer	Dermatomed (0.4 mm)	Dermatomed (0.4 mm)	0.006	0.029	4.38	3.30	Yoshimatsu et al. (2017)
Dextran	50% ethanol in Phosphate Buffered Saline	Dermatomed (500–600 μm)	Dermatomed (500–600 μm)	0.38	0.26	17.6	30.2	Dave et al. (2016)

Future efforts and the generation of more experimental data will help to elucidate the translational relationship of drug absorption between pig and human skin, potentially enabling robust interspecies extrapolation of IVPT data. This could facilitate the acquisition of more accurate absorption parameters while reducing experimental costs.

### 7.3. Limited model development for special populations

A final issue warranting attention is that the coverage of physiological factors remains incomplete in current skin PBPK studies. Specifically, patients with skin lesions, as well as pediatric and geriatric populations, which are the most frequent users of TDS, exhibit significant alterations in skin physiology. However, the incorporation of relevant physiological parameters for these groups remains scarce, and their subsequent impact on PK following TDS administration is poorly understood. Future research should prioritize the development and extensive validation of skin PBPK models for these special populations to ensure robust PK predictions.

## 8. Developmental trends in ‘digital twin’ skin PBPK models

Current skin PBPK models, whether designed for healthy individuals or special populations, primarily rely on average physiological parameters (e.g. mean SC thickness) from databases. This population-based approach struggles to capture significant physiological variations between individuals, which hinders precise individualized PK predictions. Recently, the rapid development of high-resolution ‘optical biopsy’ technologies has offered a potential solution to this bottleneck. Specifically, non-invasive imaging techniques such as reflectance confocal microscopy (RCM, clinically known as ‘three-dimensional skin CT’) (Levine and Markowitz 2018) and line-field confocal optical coherence tomography (LC-OCT) (Matharoo et al. 2025) allow for the real-time acquisition of high-resolution cellular-level skin physiological data. These data encompass the parameters traditionally incorporated into PBPK models while providing broader quantitative and qualitative physiological information. Specifically, they include information such as SC and epidermal thickness, corneocyte morphology, hair follicle density, and skin blood flow, all obtained safely without radiation. Once multimodal skin imaging features are extracted and key physiological factors influencing TDS drug absorption are identified, these parameters can be integrated into mechanistic skin PBPK models as individualized prior parameters. These inputs facilitate the construction of a high-precision ‘digital twin’ skin model, which transitions TDS PK predictions from the population level to a more individualized level (Gonçalves et al. 2026). This advancement has the potential to improve model accuracy and may support future dynamic dose adjustments and precise personalized therapy based on real-time imaging monitoring.

## 9. Conclusions

Skin PBPK models provide critical tools for TDS products development and regulatory decision-making by integrating skin physiology, drug physicochemical properties, and formulation factors to mechanistically characterize percutaneous absorption and predict local and systemic exposure. After introducing the percutaneous absorption mechanism and the theoretical framework of skin PBPK modeling, this study compared the architectures, hypotheses, and application cases of four major commercial platforms: Simcyp, GastroPlus, Skin-CAD, and PK-Sim/MoBi, and evaluated their respective strengths and limitations.

In terms of model architecture, each platform adopts distinct structural hypotheses. Simcyp employs a mechanistic ‘bricks-and-mortar’ structure to simulate drug uptake by the SC, incorporating the effects of detailed physiological variations such as sex and anatomical site on TDS absorption, thereby increasing model complexity. Skin-CAD, as the most structurally simplified model, requires the fewest absorption parameters, thereby reducing parameter uncertainty; however, its use of a basic compartmental model for systemic distribution limits its utility in handling complex systemic disposition. GastroPlus emphasizes the mechanistic description of TDS formulation systems and is the only model to account for the evaporation of the volatile active drug. Notably, this complex process necessitates sufficient parameters for the accurate characterization of dynamic absorption. PK-Sim/MoBi serve as an open-source skin PBPK modeling platform; its absorption module is similar to Skin-CAD, whereas its systemic disposition offers physiological depth comparable to Simcyp and GastroPlus. Overall, each platform represents a different trade-off in model complexity. Researchers should select the appropriate model depth based on specific objectives: clinical decision-making generally requires complex physiological characterization, while early-stage formulation screening favors simplified models with robust parameters.

Regarding research applications, the functional focus of each software has led to differentiated application scenarios. Simcyp, supported by an extensive clinical database, has been effectively validated for PK prediction performance in scenarios with significant physiological variance, including special populations and scalp administration. Notably, its reliability is underscored by successful regulatory cases, such as supporting the FDA in reducing clinical study requirements for diclofenac gel. GastroPlus excels in formulation optimization and toxicological assessment, as its refined formulation module effectively characterizes exposure differences between dosage forms. Skin-CAD, the earliest skin PBPK software developed, is an optimal tool for IVIVE based on IVPT data due to its minimal structural parameters. It holds a prominent position in optimizing formulations and developing drug-device combinations like microneedles, though its simplified systemic disposition limits its utility in predicting DDI or PK of special populations. Finally, PK-Sim/MoBi shows high potential for pediatric modeling, and its open-source architecture allows for customized research scenarios. However, its reliability still requires validation through broader application cases.

This review outlines the common limitations of current skin PBPK research. These limitations include complex model structures lacking consensus, difficulties in acquiring key absorption parameters alongside QSAR prediction biases, and a scarcity of models for special groups such as pediatric and geriatric populations, as well as patients with skin lesions. To address these challenges, this paper proposes specific strategies. Regarding model structure, when accurate IVPT experimental data or absorption parameters are available, and when the administration site involves skin without high follicle density, a homogeneous skin assumption is advocated to enhance model robustness; otherwise, a physiologically realistic heterogeneous model represents a more suitable choice. For parameter acquisition, exploring interspecies extrapolation strategies based on porcine IVPT holds promise for mitigating QSAR prediction biases, but this approach still requires further empirical data for validation. Furthermore, expanding physiological databases and validating models for special populations is urgently needed. Additionally, this paper highlights that integrating multimodal imaging technologies to acquire individualized physiological parameters for constructing a ‘digital twin’ skin model represents a critical pathway. Ultimately, this approach holds potential to guide TDS dosing strategies from population-based predictions toward precise individualized therapy.

In summary, skin PBPK research is advancing rapidly, achieving the prediction of local and systemic exposure for various formulations and administration sites, while also enabling the prediction of clinical efficacy through integration with PD models. This technology successfully supports formulation optimization, PK prediction for specialized systems such as microneedles, and toxicity assessments for cosmetic and environmental chemicals. Most importantly, skin PBPK models are increasingly supporting regulatory decisions through virtual clinical trials. Future research must enhance skin model cross-population adaptability by incorporating physiological databases for special populations, and simultaneously accelerate the development of ‘digital twin’ skin models. These combined advancements will provide a solid foundation for precision medicine and enhanced drug development efficiency.

## Data Availability

All data analyzed in this study are included in this article as citations. Original sources are identified at the specific locations within the text and the reference list.
